# Genomic Insights into the Origin, High Fecundity and Environmental Adaptation of Hu Sheep

**DOI:** 10.1002/advs.202506492

**Published:** 2025-07-14

**Authors:** Zexuan Liu, Na Zhang, Zilong Wen, Haitao Wang, Tingting Li, Runlin Ma, Menghua Li, Daxiang Wang, Haiping Shu, Xun Huang, Jianlin Han, Yiqiang Zhao, Qiuyue Liu

**Affiliations:** ^1^ State Key Laboratory of Animal Biotech Breeding College of Biological Sciences China Agricultural University Beijing 100193 China; ^2^ State Key Laboratory of Molecular Developmental Biology Institute of Genetics and Developmental Biology Chinese Academy of Sciences Beijing 100101 China; ^3^ Yazhouwan National Laboratory Sanya 572024 China; ^4^ College of Animal Science and Technology China Agricultural University Beijing 100193 China; ^5^ Jiangsu Qianbao Animal Husbandry Co., Ltd Yancheng 224050 China

**Keywords:** coevolution, environmental adaptation, high fecundity, Hu sheep

## Abstract

Hu sheep are renowned for their adaptation to high‐temperature, humid environments and their high fecundity. Despite serving as the founder species for sheep breeding in China, the genetic mechanisms underlying these desirable traits and the breed's origin have remained unresolved. To address the longstanding challenges in understanding their adaptations and origins, comprehensive analyses utilizing genetic data is conducted from over 300 Hu sheep and ≈200 other Chinese native sheep. The study unveils the migration history of sheep and the formation of the Hu sheep in eastern China. By employing multiple screening methods, this study identifies that the *BMPR1B*, *UNC5C*, and *GRID2* synergistically contribute to the high fecundity trait in Hu sheep. The coexistence of beneficial genotypes in these genes increase the likelihood of multiple births. Notably, the results suggest a potential coevolution between environmental adaptation and reproductive traits in this breed. Overall, the study provides novel insights into the genetic origin and adaptation of Hu sheep, offering new perspectives for molecular breeding in sheep and other livestock species.

## Graphical abstract

The Hu sheep diverged from the Small‐tailed Han sheep ≈1330 years ago, and subsequently migrated from northern to warm and humid southern regions. The genes *BMPR1*B, *UNC5C*, and *GRID2* play a synergistic role in the superior reproductive traits of Hu sheep. Moreover, the results suggest a potential coevolution between environmental adaptation and reproductive traits.

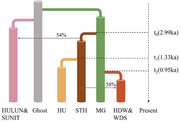

## 1 Introduction

Sheep (*Ovis aries*) were domesticated ≈10000 years ago in the Fertile Crescent and subsequently dispersed globally through human migrations.^[^ ^1^, ^2^ ^]^ The Mongolian highlands are recognized as a pivotal region for the migration of sheep to East Asia.^[^ ^3^, ^4^ ^]^ Domesticated sheep supply humans with a consistent source of agricultural products, including meat, wool, skin, and milk, and play an important role in economic, cultural, and social spheres. The history of sheep domestication in China dates back ≈5000 years ago,^[^ ^4^ ^]^ resulting in the development of over 40 populations,^[^ ^5^, ^6^ ^]^ among which the Hu sheep are one of the most renowned. The physiological characteristics of Hu sheep determine that it is a very suitable breed for intensive stall feeding. The fecundity of the Hu sheep are exceptionally high, characterized by year‐round estrus and high fecundity with an average lambing rate of 277.4%.^[^ ^7^ ^]^ It serves as the founder species for sheep breeding in China. Notably, the Hu sheep are the only population in China that has been adapted to high‐temperature and high‐humidity environments.

It is commonly accepted that Hu sheep originated from the Mongolian highlands. However, previous studies speculate this primarily through evaluating molecular distance based on a limited number of genetic loci or phenotypic similarities, lacking reliable molecular evidence of migration.^[^ ^8^, ^9^ ^]^ A recent study reconstructed the migration routes of East Asian sheep using 50K microarray data, which covered a vast geographical range from Central to East Asia. This study categorized numerous populations by large geographic regions, such as classifying Hu sheep into a broad category of northern East Asian sheep, lacking breed‐level resolution.^[^ ^4^ ^]^ At present, research at the breed level of sheep tends to focus on specific geographic regions, such as the Tibetan Plateau.^[^ ^10^ ^]^ Overall, there remains a deficiency in reliable molecular evidence regarding the origin of Hu sheep.

The domestication and breeding of sheep are influenced by a combination of artificial and natural selection, as well as environmental adaptions.^[^ ^11^ ^]^ The high temperatures and humidity throughout the Taihu Basin have contributed to the unique adaptability of Hu sheep. Most studies have explored the genetic mechanisms of environmental adaptation by comparing genomic differences between populations living in extreme environments.^[^ ^12^, ^13^, ^14^, ^15^ ^]^ Unfortunately, these studies have not effectively revealed the association between adaptation and environmental changes. In order to make use of the extensive environmental data, new research approaches such as landscape genomics are being employed. These approaches identify genetic adaptation to specific bioclimatic variables by quantifying genomic differences against bioclimatic variables.^[^ ^16^, ^17^ ^]^ However, no landscape genomics studies have been conducted on the environmental adaptations of Hu sheep to date. In addition to environmental adaptation, Hu sheep are also distinguished by their exceptional reproductive performance. Although it is widely acknowledged that the *FecB* mutation in the *BMPR1B* gene leads to the high fecundity phenotype observed in Hu sheep,^[^ ^18^ ^]^ the genetic mechanism underlying this phenomenon has not been fully elucidated. It has been observed that, despite the predominance of the *FecB* mutation in Hu sheep, the number of lambs produced by different individuals still varies.^[^ ^19^, ^20^ ^]^ This variation suggests the presence of additional genes and mechanisms beyond *FecB* that influence lambing numbers in Hu sheep.

In this study, we investigated the genetic structure and gene flow between Hu sheep and major indigenous sheep populations in China, successfully inferring the migration route of Hu sheep. To gain a deeper understanding of their environmental adaptation mechanisms, we analyzed the bioclimatic variables driving sheep differentiation and identified associated alleles. Moreover, to explore the genetic basis of high fecundity in Hu sheep, a combination of mixed linear models and multiple selection signal screening methods were employed. It is found that, in addition to *BMPR1B*, *UNC5C* and *GRID2* synergistically contribute to increased lamb production in Hu sheep.

Given the growing global demand for mutton,^[^ ^21^, ^22^ ^]^ improving lambing rates is essential for maintaining a stable and sustainable supply of high‐quality sheep meat. Identifying key genetic factors that influence high fecundity in Hu sheep provides valuable molecular markers to further improve reproductive efficiency in commercial sheep farming. Moreover, as climate change progressively transforms global agricultural systems, the development of sheep breeds resilient to evolving environmental conditions is becoming increasingly critical. Our study sheds light on the genetic basis of climate adaptation in sheep, offering insights that can inform future breeding strategies to enhance robustness and productivity in diverse environments.

## 2 Results

### 2.1 Population Structure and Demography of Hu Sheep

**Figure 1:**
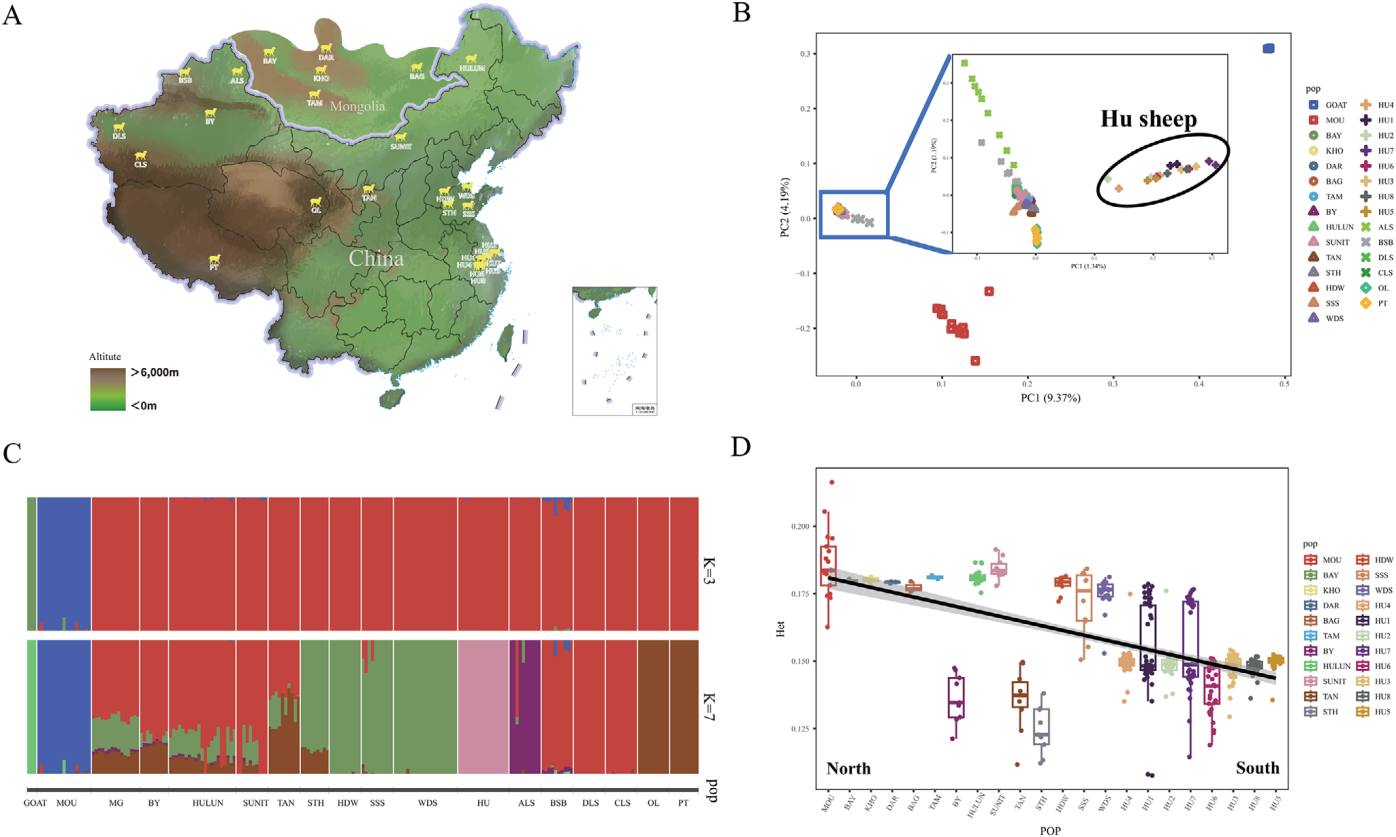
Characterization and structure analysis for sheep populations. A) Geographical distribution of populations in this study. B) PCA plot. Sheep populations from China and Mongolia are highlighted in red boxes. C) Admixture plot with K = 3 and K = 7. D) Heterozygosity distribution of Mouflon and eastern Chinese sheep populations, sorted from north to south.

In the PCA results, the outgroup goats (*Capra hircus*), the ancestral Mouflon, and the sheep from both China and Mongolia, clustered into three distinct groups (**Figure** **1**B). To better reflect the population structure of domesticated sheep in China, we redid the PCA projection exclusively for the Chinese and Mongolian populations. It reveals distinct clusters for Mongolian lineages, Tibetan lineages, and Kazakh lineages, respectively (Figure 1B), with Hu sheep positioned toward the periphery in the PCA projection. The negligible variance explained by PC1 (1.34%) indicates a lack of substantial genetic differentiation between Hu sheep and other domesticated sheep populations in China.

In the Admixture analysis with the optimal K = 3, the outgroup goat, ancestral Mouflon, and the Chinese and Mongolian populations distinctly segregated into three clusters, consistent with the PCA results (Figure 1C). At K = 7, Mongolian populations exhibited the most diverse ancestral components, confirming their ancestral status relative to domesticated sheep in China. We found distinct ancestral profiles for four populations in the Shandong region: STH, HDW, SSS, and WDS, with STH displaying a divergent ancestral composition compared to the other three groups. Moreover, at K = 7, Hu sheep exhibited distinct ancestral components, aligning with their peripheral position in the PCA. This genetic distinctiveness potentially reflects the effects of genetic drift and selective pressures during the breed's formation and development.

Despite their southernmost location in China, Hu sheep belong to the Mongolian lineage. To elucidate the genetic relationships between Hu sheep and other populations, we constructed a maximum likelihood phylogenetic tree using TreeMix. The tree topology revealed a branching order of ancestral Mouflon, followed by Xinjiang populations, Mongolian populations, Hu sheep, and certain Shandong populations. The IBS neighbor‐joining tree corroborated these phylogenetic relationships (Figure S1, Supporting Information), indicating a recent shared ancestor between Hu sheep and STH sheep. Furthermore, we observed a north‐to‐south gradient of decreasing population polymorphism (Figure 1D, β = ‐0.47, P<2.2e‐16). These findings suggest a migration pattern wherein Mongolian populations sequentially moved southward into the Yellow River and Yangtze River basins, giving rise to STH and Hu sheep, respectively. Notably, the correlation coefficient between distance from the North Pole (as measured by latitude) and lambing rate was 0.68, while the correlation coefficient for horn type was 0.20. This suggests that lambing rates increase and the prevalence of hornless traits also increases as sheep move southward.

**Figure 2:**
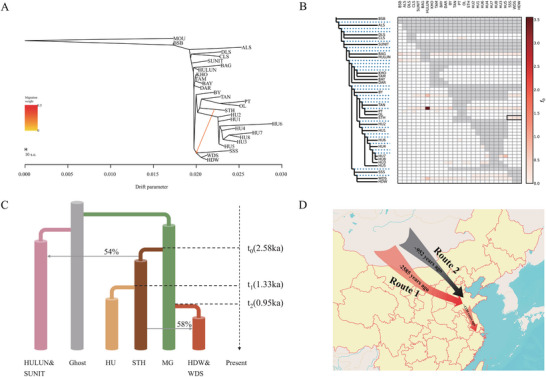
Genetic relationship and migration route inference. A) TreeMix phylogenetic tree. The arrow represents gene flow event. B) F‐branch plot for genetic exchange between populations. Darker colors indicate higher values from f_4_‐ratio; black boxes denote results involving STH and the other populations in Shandong. C) The best‐supported model of demographic history reconstruction of sheep. t represents the time of branch formation. The gray arrows indicate gene flow, and the numbers on the arrows represent the proportion of genomic contribution. D) Schematic diagram of migration routes. The red arrows indicate putative migration route 1, the black arrow indicates putative migration route 2.

According to the OptM results (Figure S2, Supporting Information), the optimal number of admixture events in TreeMix was one (**Figure** **2**A), which was the gene flow from STH to other populations in Shandong (HDW, WDS, SSS). F‐branch analysis also suggested gene flow between STH and HDW, WDS, SSS as well (Figure 2B). Together with their different ancestral components in Admixture, we proposed a second migration route: after expanding into the Yellow River basin, Mongolian sheep populations experienced gene flow with STH in the region. This process likely contributed to the formation of sheep populations such as WDS and HDW.

To validate the proposed migration routes, we performed 100 iterations of qpGraph analysis with varying parameters and obtained a stable optimal topology (score = 566.34, out‐of‐sample scores = 291.27) (Figure S3, Supporting Information). This optimal topology reveals a sequential divergence of Mongolian populations, TAN, STH, and HU, thereby corroborating the first North‐to‐South migration route of eastern Chinese sheep. Moreover, the qpGraph results indicate gene flow between STH and ancestral populations, contributing to the formation of populations such as WDS. This finding supports the existence of the second migration route.

Based on the results from qpGraph, we used fastsimcoal2 to construct a divergence model for further estimation of divergence times (Figure 2C). According to the model with the highest likelihood, the divergence between HU and STH occurred ≈1330 years ago (assuming 1.5 years per generation). For the formation of the HDW‐WDS breed, the estimated divergence time is ≈952 years ago. Additionally, the simulations revealed an admixture proportion of 58% from STH into HDW‐WDS and 54% from the ancestral population of STH and HU into HULUN‐SUNIT. These values closely align with the results from qpGraph (72% and 63%, respectively), differing by ≈10%. The two migration routes are shown in Figure 2D.

### 2.2 Adaptive Evolution Under Climatic Change

During their migration from North to South, Hu sheep experienced significant climatic changes, transitioning from a cold and dry northern environment to a warm, humid southern climate. To explore how Hu sheep adapted to these environmental changes, we investigated the adaptive evolution by integrating population genomic data with climate data.

**Figure 3:**
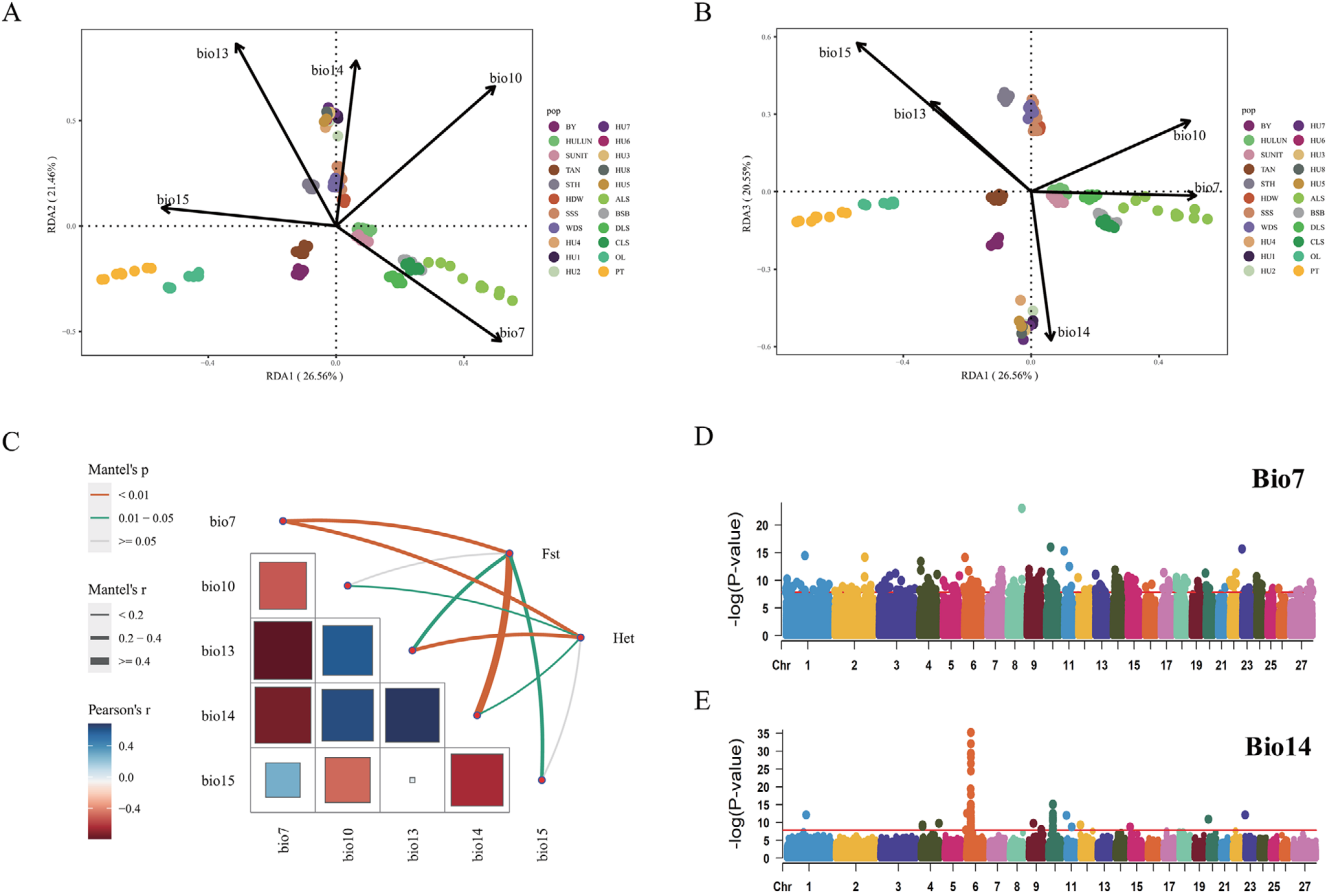
Environmental association analysis. A) RDA projection plot on RDA1 and RDA2. The dots represent the genotypic projections of each individual, and the arrows represent the bioclimatic variables. The length of the arrow represents the strength of the correlation between the bioclimatic variable and genotype. The longer the arrow, the stronger the correlation. B) RDA projection plot on RDA1 and RDA3. C) Degree of association of the five bioclimatic variables with the population Fst matrix and heterozygosity matrix. Thicker lines indicate stronger associations. The color of the lines represents the correlation p‐values: yellow, green, and gray lines represent correlation p‐values < 0.01, 0.01‐0.05, and ≥ 0.05, respectively. The heatmap represents the correlation between bioclimatic variables. D) Manhattan plot of LFMM results for bioclimatic variable BIO7, λ = 0.95. E) Manhattan plot of LFMM results for bioclimatic variable BIO14, λ = 0.96.

We observed high correlations between bioclimatic variables (Figure S4, Supporting Information). By applying a Variance Inflation Factor (VIF) criterion of <10, we retained five bioclimatic variables (BIO7, BIO10, BIO13, BIO14, BIO15), they are annual temperature range (°C), mean temperature of the warmest quarter (°C), precipitation of the wettest month (mm), precipitation of the driest month (mm), and precipitation seasonality (mm). Redundancy analysis (RDA) was conducted using the five selected bioclimatic variables as explanatory variables, and the balanced genotype data as response variables. The first three RDA components accounted for 68.57% of the variation in bioclimatic variables. Permutation tests revealed that BIO14 significantly influenced sheep in Jiangsu, Zhejiang and Shandong, while BIO7 had a greater impact on populations in Inner Mongolia and Xinjiang (p = 0.001, **Figure** **3**A,B). By grouping geographic regions, BIO14 and BIO7 were significantly correlated with heterozygosity and Fst (Figure 3C), as shown by the Mantel statistics.

We next employed Latent Factor Mixed Models (LFMM) to conduct genome‐wide association analyses for BIO7 and BIO14, aiming to identify candidate loci for environmental adaptation. For BIO7, we identified 357 associated loci (Bonferroni corrected P < 1.44 × 10^−8^) distributed across the chromosomes (Figure 3D; Table S3, Supporting Information). Annotation of these loci revealed 176 homologous genes in humans, including *UNC5C*, *BMPR1B*, and *TSHR*. Enriched KEGG pathways included circadian entrainment, lipid metabolism, and the Hippo signaling pathway (Figure S5, Supporting Information). Collectively, these loci accounted for a cumulative proportion of variance explained (PVE) of 0.43, indicating that the LFMM approach effectively captured the polygenic architecture underlying temperature adaptation. In contrast, loci associated with BIO14 exhibited significant peaks (Figure 3E, Table S4, Supporting Information), predominantly on chromosome 6 (*UNC5C*, *BMPR1B*, *PDLIM5* and *ATOH1*) and chromosome 10 (*RXFP2*). A total of 74 significant loci (Bonferroni corrected P < 1.44 × 10^−8^) collectively explained a PVE of 0.34, suggesting that genetic factors play a substantial role in adaptation to humid environments.

**Figure 4:**
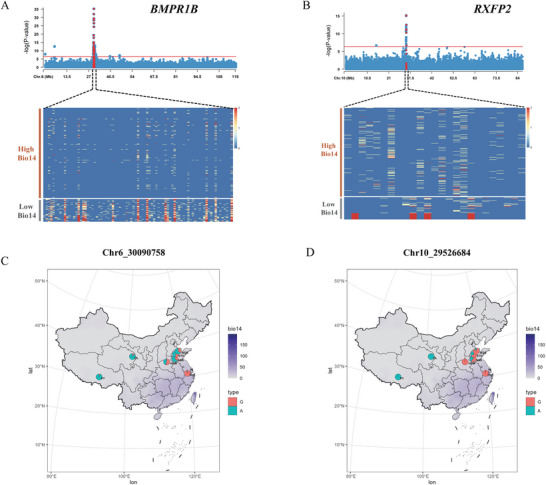
Allelic differences of candidate genes for humidity adaptation. A) Manhattan plot of LFMM results for the BIO14 bioclimatic variable in chromosome 6 region. Red dots represent loci within the *BMPR1B* gene. The heatmap shows the genotypes in the *BMPR1B* gene region for populations with high and low BIO14 values, where 0 represents homozygous for reference allele, 1 represents heterozygous and 2 represents homozygous for alternative allele. Each row of the heatmap represents one individual, and each column represents one allele. B) Manhattan plot of LFMM results for the BIO14 bioclimatic variable in chromosome 10 region. Red dots represent loci within the *RXFP2* gene. The heatmap shows genotypes in the *RXFP2* gene region for populations with high and low BIO14 values. C) Geographical distribution of allele frequencies at locus chr6:30090758 across populations. Darker color indicates higher BIO14 values which means abundant precipitation. D) Geographical distribution of allele frequencies at locus chr10:29526684 across populations.

Temperature and humidity are key bioclimatic variables differentiating northern and southern China. Hu sheep inhabits environments characterized by high temperature and humidity, while HDW, STH, SSS, WDS, OL, and PT populations live in warm and dry regions. The remaining populations reside in cold and dry conditions (Figure S6, Supporting Information). To detect allele frequency differences between populations in humid versus dry environments, we calculated Fst and π statistics. Notably, significant frequency differences were observed within the *BMPR1B* gene region between populations under dry and humid conditions (**Figure** **4**A; Figure S7, Supporting Information). For instance, at chr6:30090758, the A allele predominated in dry environment populations with a frequency of 0.67, whereas the G allele was prevalent in humid environment populations, reaching a high frequency of 0.97. The allele frequencies covary with the distribution of BIO14 (Figure 4C), suggesting that the A allele at this locus contribute to adaptation to dry environments, while the G allele facilitate adaptation to humid environments. Similarly significant allele frequency differences were observed in the *RXFP2* gene region across diverse populations (Figure 4B; Figure S8, Supporting Information). For instance, at chr10:29526684, the G allele frequency was 0.58 in dry environment populations and 0.99 in humid environment populations. Notably, in severely dry environments, such as those inhabited by OL and PT populations, the G allele frequency dropped to 0. The allele frequency at chr10:29526684 also aligned with the BIO14 gradient, suggesting that the A allele at this locus aid adaptation to dry environments, while the G allele aid adaptation to humid environments (Figure 4D). These findings suggest that the *RXFP2* and *BMPR1B* genes, exhibiting significant allele differences between humid and dry populations, may contribute to adaptation to varying precipitation levels across regions.

Overall, despite concurrent shifts in both temperature and humidity in southern regions, our findings suggest that Hu sheep demonstrate more pronounced genetic changes in response to humidity.

### 2.3 Reproduction‐Associated Genes in Hu Sheep

**Figure 5:**
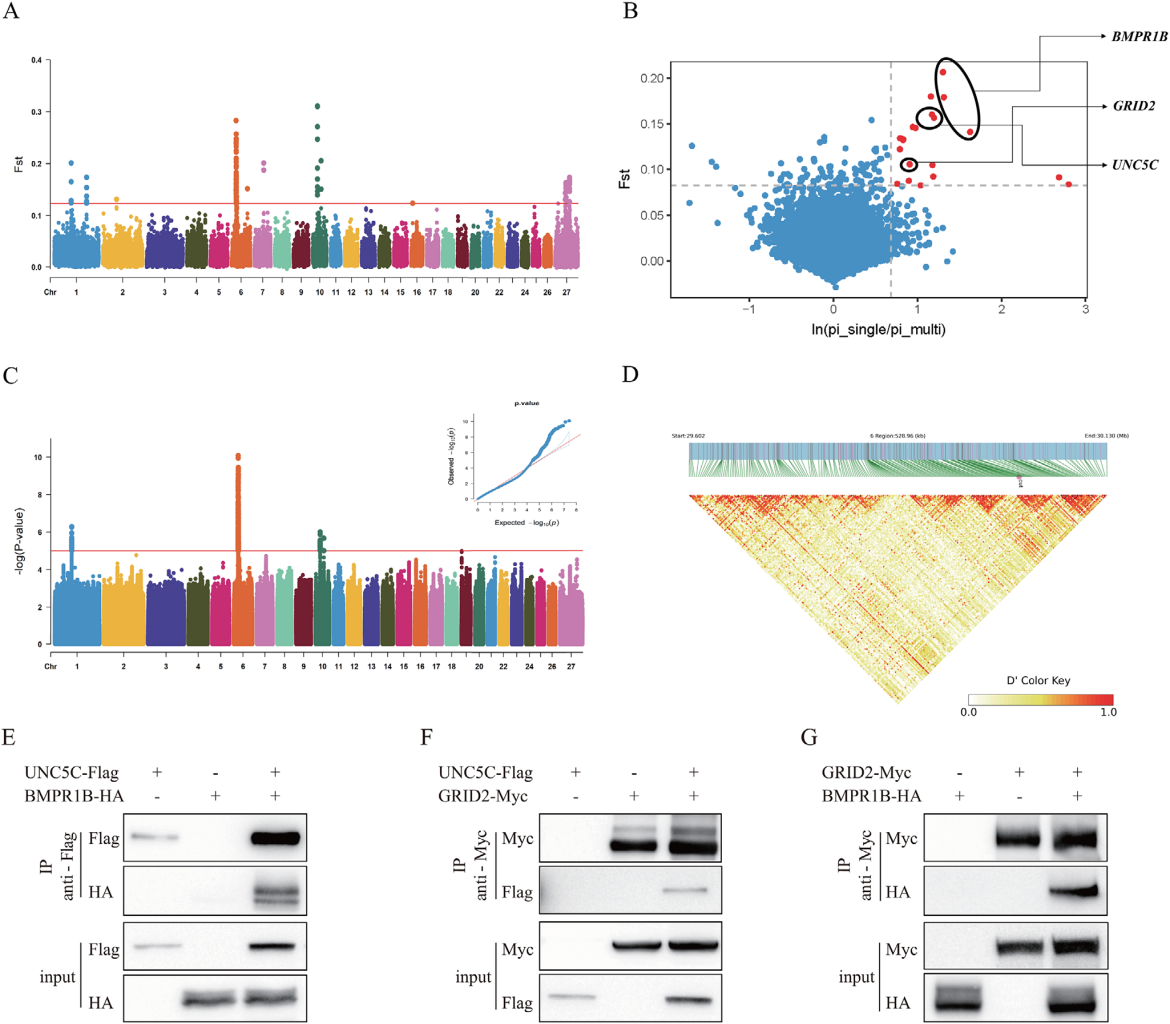
Exploring additional genes contributing to high fecundity in Hu sheep. A) Fst between Hu sheep and single‐lambing population. A threshold of top 1‰ (Fst = 0.12) was applied. B) Loci that passed the thresholds of Fst (top 0.1%) and π ratio (top 0.1% where π ratio = ln(π(single)/π(multi)) between the two populations were identified as candidate selective sweeps for high fecundity sheep. C) Manhattan plot of association analysis between Hu sheep and single‐lambing populations, λ = 1.04. Significant threshold set to P < 1 × 10^−5^. D) Heatmap shows the degree of LD in the region of *UNC5C* gene and *BMPR1B* gene. Darker colors indicate higher degrees of LD. *HEK293T* cells were transfected with constructs expressing E) *BMPR1B*‐HA and *UNC5C*‐Flag, F) *UNC5C*‐Flag and *GRID2*‐Myc, G) *BMPR1B*‐HA and *GRID2*‐Myc. Anti‐Flag or Anti‐Myc immunoprecipitates were analyzed by immunoblotting for interaction with (E) *BMPR1B*‐HA, (F) *UNC5C*‐Flag, and (G) *BMPR1B*‐HA proteins using the appropriate antibodies. Representative of two independent experiments.

Hu sheep are renowned for their high fecundity, characterized by multiple births per year and multiple lambs per litter. Previous studies have identified the *FecB* mutation in the *BMPR1B* gene as the primary genetic factor responsible for high fecundity in Hu sheep. Although the *FecB* mutation is nearly fixed in the Hu sheep population, variations in litter size among individuals suggest the existence of additional genes and mechanisms contributing to high fecundity beyond *FecB*. We compared allele frequencies between Hu sheep and single‐lambing populations, identifying two distinct peaks in the genomic regions chr6:29825001‐31575001 and chr10:29400001‐29600001 (**Figure** **5**A; Table S5, Supporting Information). To control false positives, we employed generalized mixed models to identify reliable associated signals. Compared to Fst results, the mixed model revealed a clearer signal profile, eliminating potential false positives on chromosome 27 (Figure 5C; Table S6, Supporting Information). To enhance the accuracy of genetic mapping by utilizing highly discriminative phenotypes, we sequenced 30 Hu sheep with singleton births and 17 Hu sheep with multiple births, both for at least three consecutive lambings and of comparable genetic backgrounds. Similarly, loci with Fst and π‐ratio differences in the top 0.1% between the two populations were selected as candidates (Figure 5B; Table S7, Supporting Information). Synthesizing these results, genes *BMPR1B*, *UNC5C*, and *GRID2* on chromosome 6 were consistently identified across multiple methods. Notably, *UNC5C* and *BMPR1B* were located only 9.3 Kb apart, yet linkage disequilibrium (LD) between the two regions was low (Figure 5D). Using FastEPRR, we calculated the recombination rate, where Rho = 4Ner, a parameter proportional to the recombination rate r. Our analysis revealed an average Rho value of 541.65 for chromosome 6, with the *UNC5C* and *BMPR1B* region exhibiting a notably higher average Rho of 784.66. These findings suggest that the recombination rate in this region is relatively high.

To validate the synergistic contributions of *UNC5C* and *GRID2* genes to the high fecundity in Hu sheep, logistic regression models were constructed. We selected loci with the highest Fst values in *BMPR1B*, *UNC5C*, and *GRID2* genes (chr6:30150129, chr6:30014787, and chr6:32425024, respectively) between consecutive single‐lambing and multiple‐lambing Hu sheep as explanatory variables, while the lambing phenotypes were categorized as binary response variables. We found that the multivariate model outperformed the univariate model, as evidenced by lower Akaike Information Criterion (AIC) values (**Table** **1**). The best model can be written as:

(1) $$\begin{array}{cc} logit\;\left(p\right) & =16.49\times bmpr1b\left(G\right)+16.38\times unc5c\left(C\right)\\ & \;+1.04\times grid2\left(C\right)-66.52 \end{array}$$

**Table 1:** Performance of different logistic regression models for prolificacy trait.

Model	AIC	McFadden_R^2^	p value
19.71*bmpr1b‐38.66	35.343	0.49	3.96e‐08
18.22*bmpr1b+18.13*unc5c‐71.82	34.975	0.53	5.60e‐08
16.49*bmpr1b+16.38*unc5c+1.04*grid2‐66.52	34.189	0.57	1.04e‐07
16.98*bmpr1b+14.71*grid2+16.80*unc5c‐6.83*bmpr1b*grid2‐68.35	36.189	0.57	3.98e‐07
16.96*bmpr1b+16.91*unc5c+14.71*grid2‐6.83*unc5c*grid2‐68.52	36.189	0.57	3.98e‐07
−7.51*bmpr1b‐8.09*unc5c+1.04*grid2+ 12.76*bmpr1b*unc5c‐20.64	36.189	0.57	3.98e‐07

This finding supports the hypothesis of synergistic effects of *BMPR1B*, *UNC5C*, and *GRID2* genes on the fecundity trait in Hu sheep.

By extending the study population to wild and domesticated sheep, significant allele frequency differences were observed between in the *BMPR1B* and *UNC5C* gene regions. Notably, allele frequencies of chr6:30150129_G in *BMPR1B* and chr6:30014787_C in *UNC5C* exhibited a gradient increase across different populations (Figure S9, Supporting Information). These alleles approached a frequency of 1 in Hu sheep but were nearly absent in wild populations. In contrast, no significant differences were detected in the *GRID2* gene region. Although the interaction terms among the three genes in the model were not statistically significant, functional analysis using the GeneMANIA^[^ ^23^ ^]^ mouse database revealed intricate connections among *BMPR1B*, *UNC5C*, and *GRID2* genes (Figure S10, Supporting Information). Particularly, physical interactions were observed between *BMPR1B* and *UNC5C*, suggesting potential synergistic effects. Co‐expression between *BMPR1B* and *GRID2* implied their involvement in shared gene regulatory networks or biological pathways.

To functionally validate the synergistic effects of these genes, *HEK293T* cells were co‐transfected with constructs encoding *UNC5C*‐Flag, *BMPR1B*‐HA, and *GRID2*‐Myc in pairwise combinations. Immunoblot analysis of anti‐Flag immunoprecipitates revealed that *BMPR1B*‐HA efficiently binds to *UNC5C*‐Flag, while *UNC5C*‐Flag also interacts with *GRID2*‐Myc. Notably, the interaction between *BMPR1B*‐HA and *GRID2*‐Myc was stronger than those observed in the other two pairings in live *HEK293T* cells (Figure 5E‐G).

### 2.4 Deciphering the Relationship Between Environmental Adaptation and Fecundity

**Figure 6:**
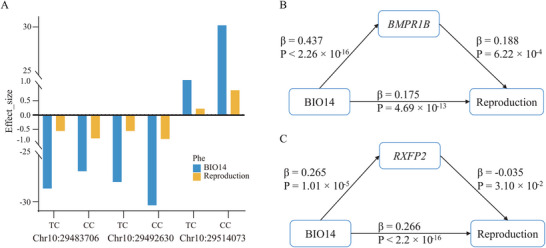
Factors influencing humidity adaptation and reproduction. A) Concordant genotypic effects for reproduction and BIO14 within the *RXFP2* gene. B) Multivariate relationships between BIO14, fecundity and frequency of SNP within *BMPR1B* using an SEM. C) Multivariate relationships between BIO14, fecundity and frequency of SNP within *RXFP2* using an SEM.

Intriguingly, *BMPR1B*, known for promoting high fecundity in sheep, exhibits signs of environmental adaptation. We also identified overlapping beneficial genotypes within the *RXFP2* gene, associated with both reproductive phenotypes (as revealed by GWAS) and humidity adaptation (as indicated by LFMM analysis) (**Figure** **6**A). These findings suggest a potential coevolution between environmental adaptation and high fecundity in Hu sheep.

To explore the multiple relationships between precipitation, fecundity and genetic factor, a structural equation model (SEM) is introduced to show the influencing pathways. The SEM indicated that multiple‐lambing is positively influenced by BIO14 either directly (p‐value < 0.05) or indirectly (p‐value < 0.05) through changes in genotype of *BMPR1B* or *RXFP2* (Figure 6B,C). The direct effects of BIO14 on multiple‐lambing were further confirmed under the counterfactual framework with statistical supports (p‐value < 0.05), while the mediation effect of *BMPR1B* was marginally significant (p‐value = 0.08). This suggests that the causal path connecting environmental adaptation and multiple‐lambing can be considered robust.

## 3 Discussion

### 3.1 About the Hu Sheep

Over the past 30 years, China has become the world's largest sheep producer, contributing to ≈30% of global meat output (National Bureau of Statistics of China, https://data.stats.gov.cn/english/easyquery). Statistics show that over 70% of sheep farms in China have now adopted fully stall‐fed or semi‐stall‐fed systems, reflecting a shift toward industrialization. The physiological characteristics of Hu sheep make them particularly well‐suited for intensive stall feeding. Hu sheep are currently one of the primary breeds promoted by the Ministry of Agriculture and Rural Affairs of China and are widely used as the maternal breed in the development of new meat sheep breeds.

### 3.2 Migration Routes of Sheep in Eastern China

In this study, we conducted population structure analysis on 29 sheep populations, revealing close genetic relationships between Chinese indigenous populations and Mongolian populations (Figure 1). We inferred a southward migration of sheep from the North to the Yangtze River basin, traversing the Yellow River basin in Shandong where the STH population formed. In the *Tan Zhi*, there are early records of Hu sheep: “Anji and Changxing, near Jiangdong, have many white sheep. Nowadays, in rural areas, there are hornless sheep known as Hu sheep”. This description highlights not only their early distribution in the south but also a shift in breeding practices from free‐range grazing to enclosed farming. During the migration process, a small portion of the sheep population moved south. The founder effect reduced genetic variability, while genetic drift further decreased diversity by randomly fixing or losing certain alleles, resulting in reduced genetic variability and increased differentiation among populations.

In the phylogeny and qpGraph results, Hu sheep and STH sheep were identified as sister populations (Figure 2A,C). Historical records indicate that STH sheep emerged in the Yellow River basin ≈900 years ago (mid‐Song Dynasty), while Hu sheep migrated to the Yangtze River basin ≈880 years ago (following the Song Dynasty's capital relocation to Lin'an).

Intriguingly, despite their shared geographical location in Shandong, STH and the other three populations (HDW, WDS, and SSS) exhibited significant genetic differentiation and were located on different branches of the phylogenetic tree. Based on qpGraph results, we inferred that Mongolian populations dispersed to the Yellow River Basin and subsequently experienced gene flow with STH, contributing to the formation of HDW, WDS, and SSS populations. Historical records indicate that the domestication of WDS, HDW, and SSS occurred ≈500 years ago,^[^ ^7^, ^24^ ^]^ considerably later than the domestication of STH sheep. Coalescent simulations tend to yield slightly earlier estimates but remain relatively close to historical data. This discrepancy can be explained by the fact that phenotypic fixation within populations typically occurs after the initial divergence, meaning historical records often capture later stages of the process. This temporal sequence supports the hypothesis that migration route 2 occurred subsequent to migration route 1.

Collectively, our findings bridge a gap in understanding the sheep migration patterns in East China. By leveraging high‐throughput data and employing multiple analytical methods, we have provided robust evidence for the origins of Hu sheep and elucidated the migration history of sheep populations in this region.

### 3.3 Reproduction‐Associated Genetic Factors in Hu Sheep

Hu sheep exhibit exceptional reproductive performance, characterized by year‐round estrus and high fecundity. Consistent with previous studies, we have confirmed the *BMPR1B* gene as a highly significant genetic factor influencing fecundity.^[^ ^25^ ^]^ However, notable variations in lambing numbers persist even among individuals homozygous for *FecB*.^[^ ^19^, ^25^, ^26^ ^]^ In TAN sheep and their hybrids *FecB* copy number has demonstrated no discernible effect on lambing rates.^[^ ^27^, ^28^ ^]^ Through the application of multiple analytical methods and the integration of two independent datasets, we have concurrently identified three genes of interest: *BMPR1B*, *UNC5C*, and *GRID2*. Our findings provide a more comprehensive understanding of the genetic basis for high fecundity in Hu sheep, as well as valuable theoretical guidance for further optimization of reproductive performance in Hu sheep breeding programs.

*UNC5C* plays a crucial role in neurodevelopment, functioning as a pro‐apoptotic molecule in concert with its ligand netrin‐1 to regulate axonal migration.^[^ ^29^, ^30^ ^]^ While it was extensively studied in human diseases like cancer and Alzheimer's, recent investigations have linked *UNC5C* to reproductive conditions such as polycystic ovary syndrome.^[^ ^31^, ^32^ ^]^ Research has shown that *UNC5C* reduces germ cell apoptosis in *UNC5C* mutant male mice.^[^ ^33^ ^]^ In Holstein cows, *UNC5C* is associated with conception rates and may influence preimplantation embryonic development.^[^ ^34^ ^]^ Moreover, genes in the apoptotic pathway have been linked to endometriosis.^[^ ^35^ ^]^ These findings collectively suggest that the *UNC5C* gene, associated with apoptosis, may play a significant role in animal reproductive function. In sheep, recent genomic differential analyses by Fst and XP‐EHH have indicated that *UNC5C* may be associated with reproductive traits,^[^ ^36^ ^]^ though more comprehensive studies are still needed. Despite its proximity (9.3 Kb) to *BMPR1B*, *UNC5C* is not in strong LD with *BMPR1B* in our study population, suggesting an independent role in the reproductive traits of Hu sheep.

The *GRID2* gene plays a critical role in the central nervous system, regulating functions such as learning, memory, emotion, and perception.^[^ ^37^ ^]^ Research has demonstrated that *GRID2* is implicated in the proliferation and apoptosis processes of porcine oocytes.^[^ ^38^ ^]^ In Dazu black goats, *GRID2* was identified as a potential influencer of lambing number through analysis of individuals of extreme phenotypes.^[^ ^39^ ^]^ Genome‐wide association studies have further revealed associations between *GRID2* and lactation persistency in Mediterranean dairy sheep,^[^ ^40^ ^]^ as well as lambing number in East Hu crossbred sheep.^[^ ^41^ ^]^ In STH, *GRID2* exhibited differential expression in pituitary tissues during the follicular and luteal phases of individuals with varying *FecB* genotypes. This suggests a role in mediating the release of reproduction‐related hormones to regulate ovulation and follicular development.^[^ ^42^ ^]^

There is increasing evidence that the mammalian ovary is connected to both the central and peripheral nervous systems, functioning in tandem with the hypothalamic‐pituitary‐gonadal (HPG) axis.^[^ ^43^ ^]^ The autonomic nerves reach the ovary through two main pathways: the superior ovarian nerve (SON), which runs within the suspensory ligament, and the ovarian plexus nerve (OPN), which travels alongside the ovarian artery and vein.^[^ ^43^, ^44^ ^]^ Both neural and endocrine signals converge to regulate steroidogenesis and ovarian follicular development. *UNC5C* is a member of the UNC‐5 family of netrin receptors. Netrins are secreted proteins that play a key role in guiding axon extension and cell migration during neural development. *GRID2* is part of the ionotropic glutamate receptor family, which includes the primary excitatory neurotransmitter receptors in the mammalian brain. Our preliminary research indicates that *BMPR1B* mutations influence ovulation rates in sheep,^[^ ^45^ ^]^ and this process is likely regulated by genes involved in the central or peripheral nervous systems.

### 3.4 Environmental Adaptation of Hu Sheep

Hu sheep underwent substantial environmental changes along their migration routes, ultimately adapting to climates markedly different from their original habitats. Climate change serves as a catalyst for natural selection.^[^ ^46^ ^]^ Under the current global warming trends, understanding the genetic mechanisms underlying thermal adaptation in Hu sheep provides a crucial molecular foundation for breeding programs and enhancing animal productivity. As a complex trait, heat tolerance adaptation typically involves multiple genes, a pattern observed in insects,^[^ ^47^ ^]^ Ethiopian sheep,^[^ ^48^ ^]^ and Mediterranean dairy sheep.^[^ ^49^ ^]^ KEGG enrichment analysis revealed several pathways related to energy metabolism, including fat digestion and absorption, and regulation of lipolysis in adipocytes. These pathways modulate cellular responses to high‐temperature environments by redirecting energy from lipid storage to thermoregulation. For instance, among the candidate genes, *PPARGC1B* may modulate thermoregulation through its involvement in fatty acid oxidation and non‐oxidative glucose metabolism pathways.^[^ ^50^ ^]^ Additionally, *PIEZO2* ^[^ ^51^ ^]^ and *KCNQ1*,^[^ ^52^ ^]^ which are implicated in ion channel regulation, could potentially mediate cellular responses to temperature‐induced alterations in cellular membrane properties.

Traditionally, studies on humidity adaptation have primarily focused on drought tolerance.^[^ ^53^ ^]^ However, in the case of Hu sheep, adaptation to humid environments warrants more attention. We identified 74 loci significantly associated with precipitation, involving 15 genes including *BMPR1B*, *RXFP2*, and *UNC5C*. High temperatures and humidity increase the likelihood of disease and parasite proliferation.^[^ ^54^ ^]^ *UNC5C* stood out due to its role in macrophage chemotaxis and T‐cell activation, thereby enhancing disease resistance.^[^ ^55^ ^]^ Similarly among the candidate genes, *SYK* modulates inflammatory responses,^[^ ^56^ ^]^ while *IL32* mediates immunomodulatory functions via cytokine signaling within immune reactions.^[^ ^57^ ^]^ Furthermore, the polledness‐associated *RXFP2* gene in Hu sheep confers enhanced disease resistance through dual mechanisms: preventing horn‐induced tissue damage and infection risks while enabling energy redistribution to critical biological functions including immune regulation, reproductive efficiency, and foraging capacity.^[^ ^58^, ^59^ ^]^ The *DSC1* gene generates adhesive proteins that are involved in intercellular junctions and enhance skin barrier function.^[^ ^60^ ^]^ Interestingly, we found that the allelic frequencies of *UNC5C*, *BMPR1B*, and *RXFP2* genes among populations covaried with precipitation levels. It is important to note that complex traits are influenced by numerous genes, with peripheral genes indirectly affecting traits through interactions with core genes and their roles in different physiological processes. Although current literature does not directly link these genes to wet adaptation, they may be associated with peripheral or indirect functions related to humid environments.

Overall, based on the candidate loci identified, genetic marker‐assisted selection can be employed to develop breeds with enhanced environmental adaptability. This will also enable more precise delineation of species‐specific habitat ranges and inform translocation strategies for conservation and genetic resource management, with the ultimate goal of mitigating the potential adverse impacts of global climate change on biodiversity and agricultural productivity.

### 3.5 Coevolution Between Environmental Adaptation and Reproductive Traits

Temperature profoundly influences mammalian reproductive functions, involving spermatogenesis, oocyte development and maturation, early embryonic development, fetal and placental growth, and lactation.^[^ ^61^ ^]^ It is well established that elevated temperatures can induce heat stress in sheep, adversely affecting their productive and reproductive capabilities.^[^ ^62^, ^63^ ^]^ KEGG enrichment analysis of temperature‐adapted genes identified several reproduction‐related pathways, including “Hippo signaling pathway” that is associated with ovarian development, “WNT signaling pathway” that regulates mammalian reproductive development,^[^ ^64^ ^]^ “Synthesis and degradation of ketone bodies” that is crucial for maintaining pregnancy, and “One‐carbon pool by folate” that is linked to germ cell proliferation.^[^ ^65^ ^]^ Among the temperature‐adapted candidate genes, *BMPR1B* and *TSHR* are critical for reproduction. *BMPR1B* is widely recognized as the primary effector gene for high fecundity in Hu sheep. *TSHR* catalyzes the synthesis of cAMP, playing a pivotal role in the release of follicle‐stimulating hormone and luteinizing hormone as secondary messengers.^[^ ^66^ ^]^ Notably, *TSHR* has been positively selected in sheep^[^ ^15^, ^67^ ^]^ and chickens^[^ ^14^ ^]^ inhabiting hot regions. In sheep reproduction, *TSHR* is instrumental in metabolic regulation and photoperiod control, promoting heat tolerance responses in pregnant ewes.^[^ ^68^ ^]^

Previous research has indicated that sheep experience significant heat stress responses only when temperatures exceeding 32 °C or even 36 °C.^[^ ^68^ ^]^ In the region where Hu sheep are located, the average temperature during the hottest month ranges from 24.4 to 30.8 °C, which is below the heat stress threshold. Our findings suggest that adaptation to moderate warming may actually enhance the reproductive performance of Hu sheep. This concept aligns with studies on other species. For instance, in Drosophila, varying intensities of heat stress have been shown to produce different evolutionary outcomes, with gradual warming leading to increased fecundity and viability.^[^ ^69^ ^]^ Similarly, the bruchid beetle demonstrated a 51% higher fecundity at 27 °C compared to 17 °C.^[^ ^70^ ^]^ It is understandable that lower latitudes tend to have more uniform photoperiods and higher temperatures, allowing animals easy to find suitable environments for reproduction throughout the year, thus exhibiting year‐round estrus. This phenomenon may establish a connection between heat adaptation and high fecundity in Hu sheep. Mammalian reproduction is a physiologically demanding process with a high energetic cost to the mother.^[^ ^71^ ^]^ In warmer environments, abundant precipitation ensures a consistent forage supply, thereby reducing survival stress for Hu sheep and providing the necessary energy for reproduction. Moreover, enhanced immunocompetence, which addresses the challenges of disease transmission associated with high temperatures and humidity, further contributes to the potential for high fecundity in Hu sheep. Similar adaptive changes can be observed in white‐tailed deer. For instance, white‐tailed deer in the United States face few natural predators, abundant food resources, and consequently, higher reproductive rates.^[^ ^72^ ^]^ Research has also documented an intriguing reproductive pattern wherein pregnancy rates are elevated among younger individuals with comparatively smaller antler development (1‐year‐old).^[^ ^73^ ^]^ This phenomenon suggests that reduced competition has led to altered reproductive dynamics, rendering antler size no longer being a key factor for reproductive success. Given that Hu sheep are the sole indigenous sheep in China adapted to high temperature and humidity conditions, opportunities for cross‐validation with other populations are limited. Therefore, additional functional experiments are essential to substantiate this hypothesis.

In addition to the current analyses, further research is needed to validate the functional roles of the identified loci. CRISPR‐based functional validation could help confirm their direct effects on fecundity and environmental adaptation. Moreover, GWAS in larger and more diverse sheep populations, combined with environmental data, would further elucidate genotype‐environment interactions.

## 4 Conclusion

In this study, we integrated genetic data and environmental data to investigate the origin of the Hu sheep and identify environmental adaptation loci during its migration route. We also localized loci associated with reproductive traits in Hu sheep using various methods. Furthermore, we discovered that there might be a coevolution between environmental adaptation and reproductive traits in Hu sheep. These insights are particularly relevant for enhancing sheep productivity in the face of climate change and refining breeding strategies to optimize adaptability across diverse ecological conditions. Overall, our study provides novel insights into the origin and adaptation of Hu sheep, offering a fresh perspective for molecular breeding in livestock species. These findings contribute to the advancement of genetic improvement strategies, supporting global food security and the sustainability of animal husbandry.

## 5 Experimental Section

### Sample and Genetic Data Collection

The genotypic data analyzed in this study came from 465 individuals from 15 Chinese native sheep populations and Asian Mouflon (MOU, 17). Specifically, eight Hu sheep populations were all collected from national core breeding or genetic resource breeding farms as follows: Jiangsu Province: Suzhou (HU1, 44), TaiCang (HU2, 26), Yancheng (Hu sheep with single births and multiple births, 48); Zhejiang Province: Xiaoshan District of Hangzhou (HU3, 53), Changxing District of Hangzhou (HU4,46 and HU5, 20), Nanxun District of Hangzhou (HU6, 29), Yuhang District of Hangzhou (HU7, 32), Lin'an District of Hangzhou (HU8, 22). Other Chinese native sheep populations included in this study were: Small‐tailed Sheep (STH, 9), Wadi Sheep (WDS, 20), Sishui Fur Sheep (SSS, 10), Large‐tailed Sheep (HDW, 10), Tan Sheep (TAN, 10), Hulunbeier Sheep (HULUN, 21), Sunit Sheep (SUNIT, 10), Prairie‐Type Tibetan Sheep (PT, 9), Ola Tibetan Sheep (OL, 10), Dolang Sheep (DLS, 10), Cele Black Sheep (CLS, 10), Bayinbruk Sheep (BY, 9), Bashbay Sheep (BSB, 10), and Altai Sheep (ALS, 10). The Mongolian sheep populations (MG) included: Tamir (TAM, 3), Khotont (KHO, 3), Darkhad (DAR, 3), Bayad (BAY, 3), and Barga (BAG, 3). According to lineage classification, the Mongolian lineages include HU, STH, WDS, SSS, HDW, TAN, HULUN, SUNIT, BY, CLS and MG. Tibetan lineages include PT and OL, while Kazakh lineages include DLS, ALS, and BSB. The sampling locations of the populations are shown in Figure 1A. Three goats were also included and served as the outgroup. Furthermore, 30 individuals of Hu sheep with singletons for three consecutive lambings and 17 individuals with multiple births for three consecutive lambings were collected from Jiangsu Qianbao Animal Husbandry Co., Ltd (Yangcheng, Jiangsu). Sample details can be found in Table S1 (Supporting Information). Phenotypes of Hu sheep with single births and multiple births can be found in Table S2 (Supporting Information).

All experimental protocols were conducted according to the guideline of Animal Advisory Committee at Institute of Genetics and Developmental Biology, Chinese Academy of Sciences (approval No. AP2022015‐C1).

### DNA Extraction and Sequencing

SUNIT, HULUN and all Hu sheep except HU1 (PRJCA025044) were sequenced in this study (PRJCA029248). The collected jugular vein blood was initially stored at −80 °C. Subsequently, sample testing was performed to assess concentration, sample integrity, and purity. Samples meeting the quality criteria were then subjected to genomic DNA fragmentation and size selection. The fragments were then end‐repaired, adenylated to add an “A” tail, and ligated with specific adaptors. The resulting products were amplified via ligation‐mediated PCR. PCR products were purified, and the library was amplified using phi29 to create DNA nanoballs (DNBs). These DNBs were loaded onto a patterned nanoarray, and paired‐end 100/150 base reads were generated through combinatorial Probe‐Anchor Synthesis (cPAS). Sequencing was conducted on DNBSEQ‐T7 platform, yielding 150 bp paired‐end reads with an average sequencing depth of 9.08X.

Remaining sample data were from public databases. The goat population was derived from project PRJNA941958. The MG population was from PRJNA645671. DLS, WDS, HDW, SSS, CLS, BSB, ALS, and MOU were from PRJNA624020. TAN, STH, PT, OL, and BY were from SRP066883. All the codes were available at github (https://github.com/liuze955/zexuanLiu_et_al._2025).

### SNP Calling and Filtering

Both read alignment and variant calling were performed by GTX‐One variants caller, which was a commercial FPGA‐accelerated version of BWA and GATK.^[^ ^74^ ^]^ The gVCFs for each sample were generated by the GTX‐One variants caller directly from Fastq files based on the ARS‐UI_Ramb_v2.0 reference genome, using the command gtx wgs. Joint variant calling was conducted on all gVCF files to generate a multi‐sample VCF file using gtx gi and gtx joint commands from GTX.One, corresponding to the GenomicsDBImport and GenotypeGVCFs from GATK. The SNP loci were extracted for the VCF file using GATK's SelectVariants command, followed by quality control using the VariantFiltration command, with parameters set to QD < 2.0, QUAL < 30.0, SOR > 3.0, FS > 60.0, and MQ < 40.0. This process resulted in 127896853 SNP loci. Subsequently, further filtering was performed using VCFtools^[^ ^75^ ^]^ to retain bi‐allelic loci (–min‐alleles 2 –max‐alleles 2), remove loci with a deletion rate greater than 10% (–max‐missing 0.9), and exclude individuals with a deletion rate exceeding 20% (–missing‐indv). Based on the filtered VCF file, Beagle was employed to phase the genotype without a reference panel with default settings. Plink^[^ ^76^ ^]^ was then utilized for LD filtering with the parameter “–indep‐pairwise 50 10 0.2”. Finally, loci with a minor allele frequency (MAF) less than 0.01 were removed using VCFtools with the parameter “–maf ”, resulting in a final set of 465 individuals with 3475647 SNPs.

### Population Structure

As both PCA and Admixture analyses can be significantly influenced by sample size,^[^ ^77^, ^78^ ^]^ it used balanced sample sizes across populations to reduce potential biases. Each Hu sheep population was sampled by two individuals, thereby maintaining a comparable number of 16 Hu sheep to other populations. Consequently, a total of 209 individuals were utilized for PCA and Admixture analyses. The first two Principal Components were calculated using GCTA software, which initially computes the genomic relationship matrix (GRM). This matrix represents the pairwise genetic relatedness between individuals based on their genotypic data.^[^ ^79^ ^]^ Similarly, PCA analyses were conducted independently for subsets of domesticated sheep for Chinese and Mongolian populations.

Unsupervised Admixture analysis,^[^ ^78^ ^]^ was performed on the genotype data of all samples, using predefined genetic clusters (K) ranging from 2 to 7, with 10 replications for each K. The optimal K was determined according to the smallest cross‐validation (CV) error.

### Genetic Diversity and Recombination Rate

VCFtools was used to compute heterozygosity statistics for individuals using the parameter “–het”. Population polymorphisms were determined using the parameter “–window‐pi 50000 –window‐pi‐step 25000”. LD of regions around candidate gene was calculated and visualized using LDBlockShow software.^[^ ^80^ ^]^ The FastEPRR_VCF series of functions in the FastEPRR^[^ ^81^ ^]^ tool were used to calculate recombination rates, with a window size of 50 kb.

### Phylogenetic and Gene Flow Analysis

The TreeMix software^[^ ^82^ ^]^ employs the maximum likelihood method to infer genetic relationships between populations, while accounting for potential gene flow. The number of gene flow events (m) was preset between 1 and 10, with each value replicated 10 times. MOU was utilized as the root of the phylogenetic tree in the TreeMix analysis. To identify the optimal m, the TreeMix results were evaluated using the R package OptM.^[^ ^83^ ^]^ The largest ΔM, corresponding to m = 1, was selected as the optimal m and plotted using plotting_funcs.R in TreeMix.

The f_4_‐ratio was a quantitative measure of the proportion of admixture from different source populations in an admixed population.^[^ ^84^ ^]^ The F‐branch method combined phylogenetic tree and the f_4_‐ratio values to reveal evolutionary history and genetic exchanges between different populations. The phylogenetic tree produced by TreeMix was used as an input to calculate the f_4_‐ratio for each topology using the Dsuite software.^[^ ^85^ ^]^ The resulting F‐branch matrix was visualized using dtools.py.

The qpGraph module in Admixtools2^[^ ^86^ ^]^ uses f_3_ statistic and the admixture graph to find edge weights that minimize the difference between the predicted f_3_ statistic and the observed f_3_ statistic. To verify the inferred migration route of sheep in eastern China, it first performed 100 independent graph searches for each number of admixture events (1‐3) using the find_graphs function. Graph comparisons were performed for the top best‐scoring graphs, and the optimal model was selected. In addition, it also compared out‐of‐sample scores, which allows a fair comparison of models with varying complexity due to different numbers of admixture events.

Fastsimcoal2^[^ ^87^ ^]^ enables the simulation of genetic and genomic data under complex evolutionary models using a fast sequential Markov coalescent framework. The estimation of demographic parameters from complex evolutionary models by fastsimcoal2 was based on the site frequency spectrum. To simplify the model, it combined HULUN and SUNIT into one group and HDW and WDS into another. Using easySFS,^[^ ^88^ ^]^ it calculated the site frequency spectrum. Subsequently, it estimated the maximum likelihood values for scenarios derived from qpGraph, assuming a ghost population as the common ancestor of the studied populations. A total of 100 replicates were performed, with each replicate allowing a maximum of 400000 iterations during the maximum likelihood optimization process.

### Genotype‐Environment Association

WorldClim^[^ ^89^ ^]^ was a database that provides high‐resolution global climate data. The elevation data and 19 bioclimatic variables were extracted from the WorldClim version 2 using the R package raster,^[^ ^90^ ^]^ based on the geographical coordinates (latitude and longitude) of the regions where the study populations were located. These bioclimatic variables represent the mean of the historical environmental data from 1970 to 2000 at a spatial resolution of 2.5 min.

RDA was a multivariate statistical method that combines multiple regression analysis and PCA to analyze the relationship between a response variable (e.g., genotype) and sets of explanatory variables (e.g., bioclimatic variables). The RDA analysis was conducted using the R package vegan.^[^ ^91^ ^]^ To prevent multicollinearity among bioclimatic variables, the R package usdm^[^ ^92^ ^]^ was employed to filter the 19 bioclimatic variables. A criterion of VIF < 10 was applied to retain non‐redundant bioclimatic variables for subsequent analysis. As a constrained principal component analysis, RDA was also influenced by sample imbalance. Therefore, the genotype data sampled for PCA and Admixture analyses were also used as the response variable in RDA analysis.

Mantel analysis was a statistical test used to assess the correlation between two square matrices. Mantel analysis was conducted using the R package vegan^[^ ^93^ ^]^ and ggcor between the heterozygosity, Fst, and bioclimatic variables based on sheep populations. The Fst matrix between populations need no further processing, matrices for heterozygosity and bioclimatic variables were calculated between each pair of populations using Euclidean distance. Meanwhile, the Pearson correlation between bioclimatic variables was also calculated.

LFMM was used to identify SNP loci associated with environmental adaptation in the genome.^[^ ^94^ ^]^ The model was similar to the linear models used in GWAS, but in LFMM, bioclimatic variables were used as explanatory variables, and genotypes were used as response variables. The model can be written as:

(2) y=Xβ+Uv+e

where y denotes the response variable genotype matrix, X represents the explanatory bioclimatic variables, β is the vector of coefficients, U denotes the latent variable used to account for population stratification, v is the corresponding coefficients, and e is the residuals. The LFMM analysis was conducted using the R package lfmm for all bioclimatic variables and for all populations, with the parameters for population stratification set to the first three PCs. Loci with a Bonferroni‐corrected p‐value < 1.44 × 10^−8^ were considered as candidate loci for environmental adaptation.

To evaluate the degree of environmental variation explained by genetic loci, loci exceeding the significant threshold in the LFMM model were recorded. In a separated mixed model, the bioclimatic variables were included as phenotypes while an additive GRM was constructed based on genotypes of these significant loci as random effects. The PVE for these loci was further estimated using HIBLUP,^[^ ^95^ ^]^ with the model including GRMs calculated separately for significant and non‐significant loci.

### Gene Annotation and Enrichment Analysis

Genes located within 20 Kb upstream or downstream of the candidate loci were extracted as candidates. The homologous genes of these candidate genes in humans were identified using the g:Profiler website (http://biit.cs.ut.ee/gprofiler/).^[^ ^96^ ^]^ The human homologous genes were then feed into the KOBAS website (http://bioinfo.org/kobas/)^[^ ^97^ ^]^ for KEGG pathway enrichment analysis. Entries with a p‐value of less than 0.05 after Bonferroni correction were considered as significant.

### Selection Signature

Fst measures the degree of differentiation between populations based on allelic frequency. For any two populations, Fst values were calculated using VCFtools with a window size of 50 Kb and a step size of 25 Kb. Loci with the top 0.1% Fst values were considered as significant. To identify loci associated with reproduction, Fst was computed between the Hu sheep and single‐lambing sheep, which comprise the TAN, SUNIT, PT, OL, HULUN, BY, SSS, ALS, and BSB populations.

To control false positives and to obtain better interpretability, a GWAS framework was introduced to identify differentiation loci between populations. The generalized mixed linear model *logit*(*p*)  =  *X*β + *g* + *e* was fitted using the R package SAIGE.^[^ ^98^ ^]^ In this model, y represents a single‐lambing sheep or Hu sheep using binary coding, p = P (Y = Hu sheep | X, g) was the probability to be modeled; X was the genotype coded as 0, 1, and 2 as fixed effect; b was the coefficient of the fixed effect; g was the polygenic random effect as captured by GRM; and e was the residual. One advantage of SAIGE was its ability to control for bias caused by the imbalance in the binary response variables as it have more Hu sheep than single‐lambing sheep. Loci with a Bonferroni‐corrected p‐value <1 × 10^−5^ were considered as significant.

### Genetic Contribution for Litter Size

To validate the synergistic effects of loci on phenotype, a logistic regression model was constructed. This model considered the main effect of each locus and allowed for one interaction term. The model can be written as: *logit*(*p*)  =  *X*β + *Zb* + *e*, where y represents Hu sheep with singletons or Hu sheep with multiple births using binary coding, p = P (Y = Hu sheep with multiple births | X, Z) is the probability to be modeled; X is the design matrix of genotypes coded as 0, 1, and 2 for loci from *BMPR1B*, *UNC5C*, and *GRID2* (chr6:30150129, chr6:30014787, chr6:32425024). β is the coefficient vector of X, Z is an interaction term between X elements, b is the coefficient for the interaction effect; and e is the residual. The models were evaluated using the Akaike Information Criterion (AIC) and McFadden R^2^.

### Functional Validation of Synergistic Effects

The plasmids used, including pcDNA3.1. *UNC5C*‐Flag, pcDNA3.1. *BMPR1B*‐HA, and pcDNA3.1. *GRID2*‐Myc, were constructed by Sangon Biotech, Shanghai. Rabbit anti‐DDDDK (Flag tag) (AE092), anti‐Myc (AE070) mAbs, agarose beads‐conjugated anti‐Myc VHH single domain antibody (AE106), agarose beads‐conjugated anti‐DDDDK VHH Single Domain antibody (AE125), protein A/G magnetic beads (RM02915) and mouse anti‐GAPDH (AC002) were purchased from ABclonal, Inc. Mouse anti‐HA (M180) was purchased from MBL Beijing Biotech Co., Ltd.

*HEK293T* cells were maintained in Dulbecco's Modified Eagle's medium containing 10% fetal bovine serum (FBS, Gibco) and 1% penicillin‐streptomycin (Gibco). Cells were cultured in a 5% (vol/vol) CO_2_ incubator at 37 °C. H4000 (Engreen) was used for the transfection of plasmids into cells for 48 hours, according to the manufacturer's instructions.

For co‐immunoprecipitation experiments, the transfected *HEK293T* cells were lysed in 1 mL of RIPA buffer (50 mM Tris‐HCl, pH 8.0; 150 mM NaCl; 0.5 mM EDTA, 0.1% SDS, 1% sodium deoxycholate, 1% Triton X‐100 supplemented with protease inhibitor PMSF) and centrifuged at 12 000 g at 4 °C for 10 min. The supernatant was precleared with protein A/G magnetic beads and then incubated with anti‐Flag or anti‐Myc beads at 4 °C overnight with constant rotation. Immunoprecipitated samples were collected by centrifugation and washed with RIPA buffer three times. After washing, the immunoprecipitates were boiled in sample‐loading buffer for 10 min to elute the precipitated proteins, then subjected to immunoblot analysis. For immunoblot analyses, the samples were fractionated by sodium dodecyl sulfate polyacrylamide gel electrophoresis (SDS‐PAGE) and transferred onto polyvinylidene fluoride membranes (Merck). The membranes were first blocked with 5% (w/v) non‐fat milk in TBST, and subsequent immunoblot analysis was performed with the indicated primary antibodies, followed by horseradish peroxidase (HRP)‐conjugated secondary antibodies The protein bands were visualized using Pierce ECL Western Blotting Substrate (Thermo Scientific) according to the manufacturer's instructions.

### Estimation of Causal Relationship

It implemented the SEM using the R package lavaan^[^ ^99^ ^]^ to examine the multivariate relationship between variables. Specifically, it analyzed the relationships involving BIO14, reproductive trait of the population in section 5.9, where multiple‐lambing was coded as 1 and single‐lambing as 0, as well as the frequency of SNP chr6:30090758 within *BMPR1B* and chr10:29526684 within *RXFP2*. The model was as follows:

(3) reproductivetrait=a∗BIO14+c∗freq

(4) freq=b∗BIO14

The indirect influence of BIO14 on reproductive trait through the allele frequency was calculated, with a path coefficient represented as b*c. The model was fitted using the maximum likelihood estimation method. The significance of the path coefficients was tested via standard error estimates and t‐values. The significance level was set at 0.05.

In addition to the regression‐based SEM framework implemented using lavvan, it also investigated the causal relationship between BIO14 and reproductive traits by conducting a mediation analysis based on the counterfactual framework using the R package Mediation.^[^ ^100^ ^]^ In the mediation analysis, BIO14 served as the independent variable, the dependent variable as the reproductive trait, and the frequency of SNPs as the mediator variables.

## Conflict of Interest

The authors declare no conflict of interest.

## Supporting information



Supporting Information

Supporting Information

## Data Availability

The data that support the findings of this study are openly available in china national center for bioinformation at https://ngdc.cncb.ac.cn/gsub/submit/bioproject/PRJCA029248, reference number 29248.
