# Supplementary material for: Genomic Insights into the Origin, High Fecundity and Environmental Adaptation of Hu Sheep
Source: Adv Sci (Weinh). 2025 Jul 14;12(37):e06492. doi: 10.1002/advs.202506492 (PMC12499457; doi:10.1002/advs.202506492)
Supplement: Supplementary file 1 — Supporting Information [file ADVS-12-e06492-s001.docx]

Supplementary Figures


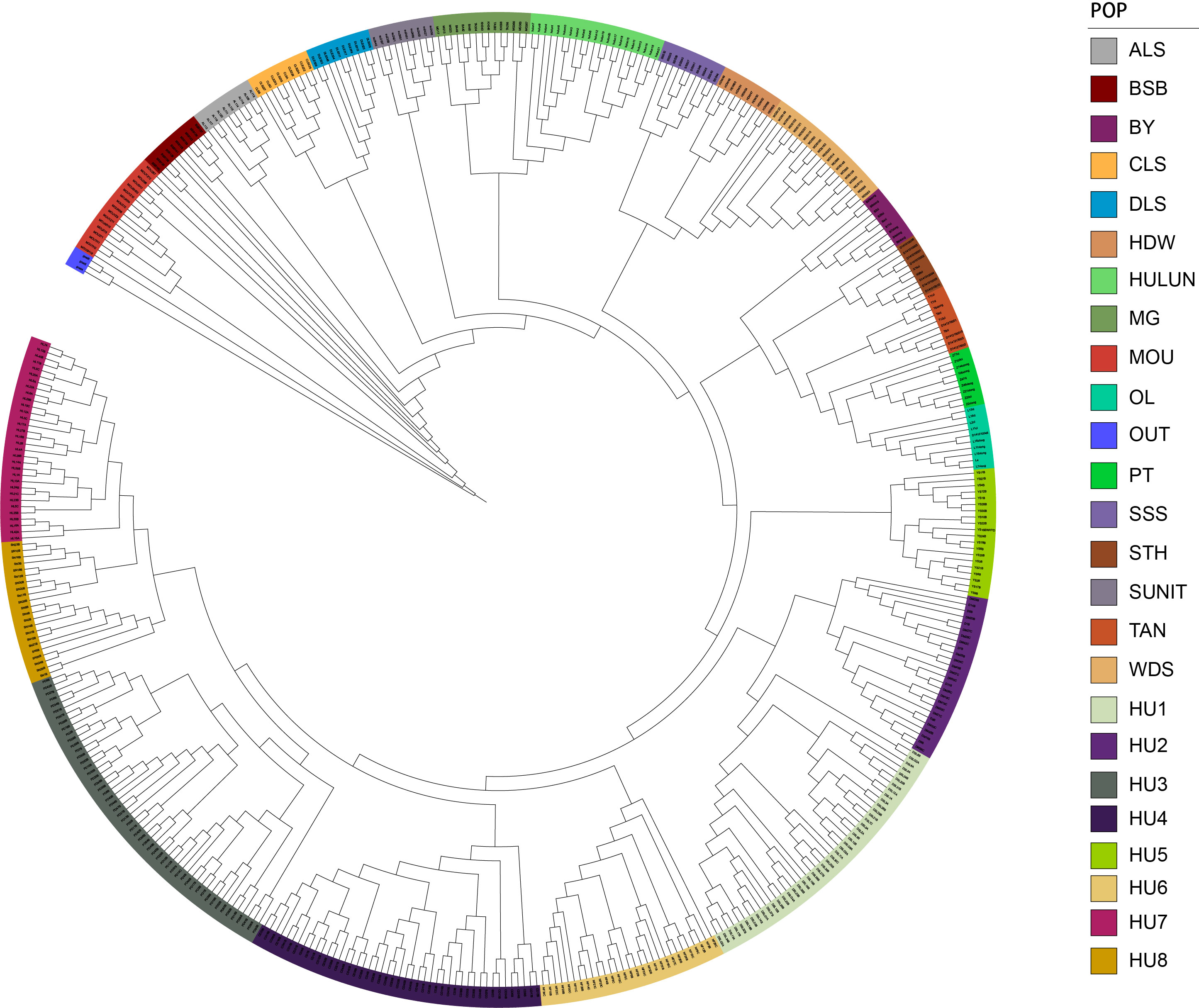


**Figure S1**. IBS-NJ tree. The outgroup is ibex goat. The tree topology revealed a branching order starting with ancestral Mouflon, followed by Xinjiang populations, Mongolian populations, Shandong populations and Hu sheep. This order is consistent with the likelihood tree constructed by TreeMix.


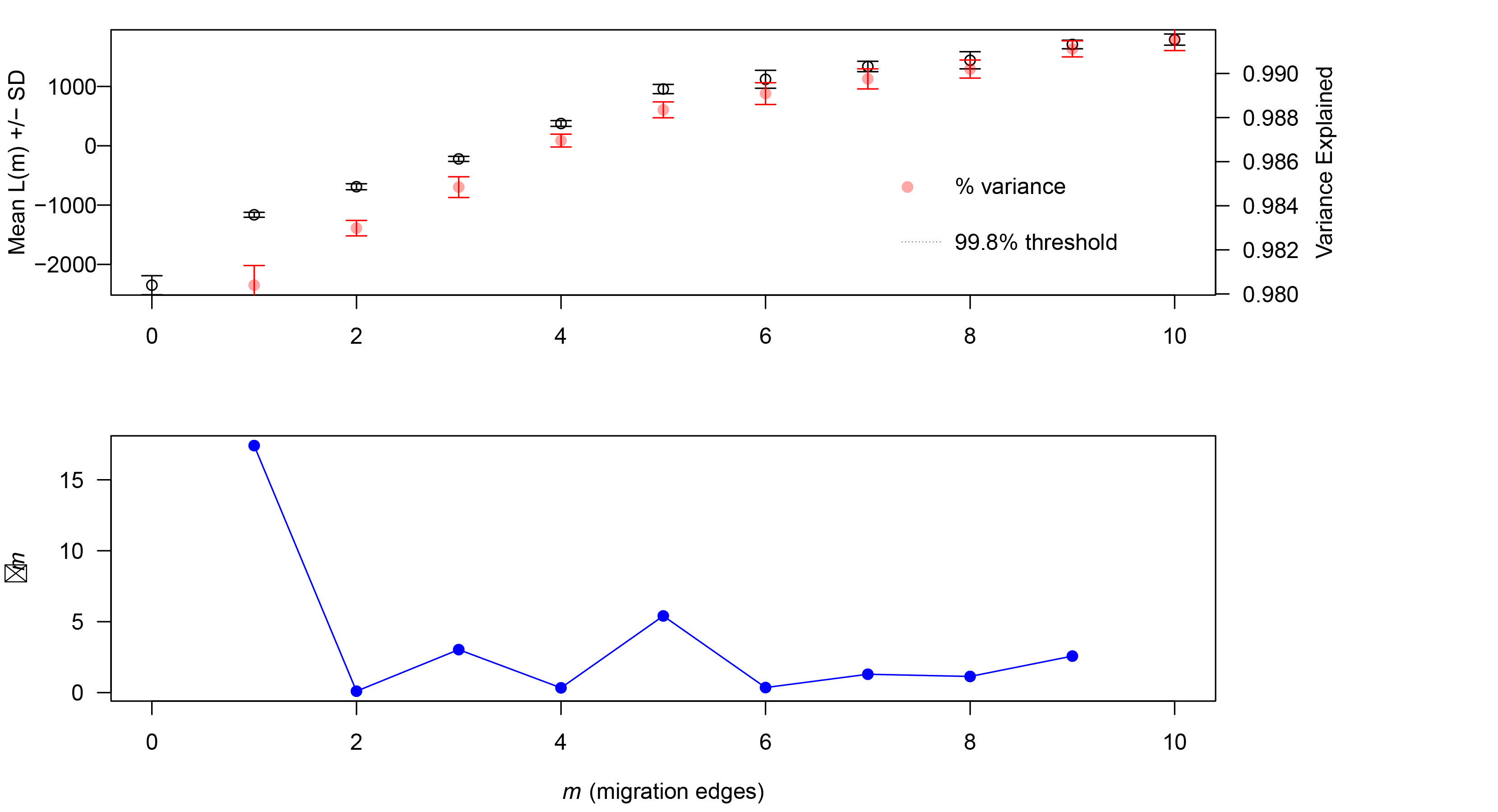


**Figure S2**. Result OptM model fitting. The result of OptM evaluated different m results of TreeMix, demonstrating the best fit of TreeMix for m = 1 with the largest Δm.


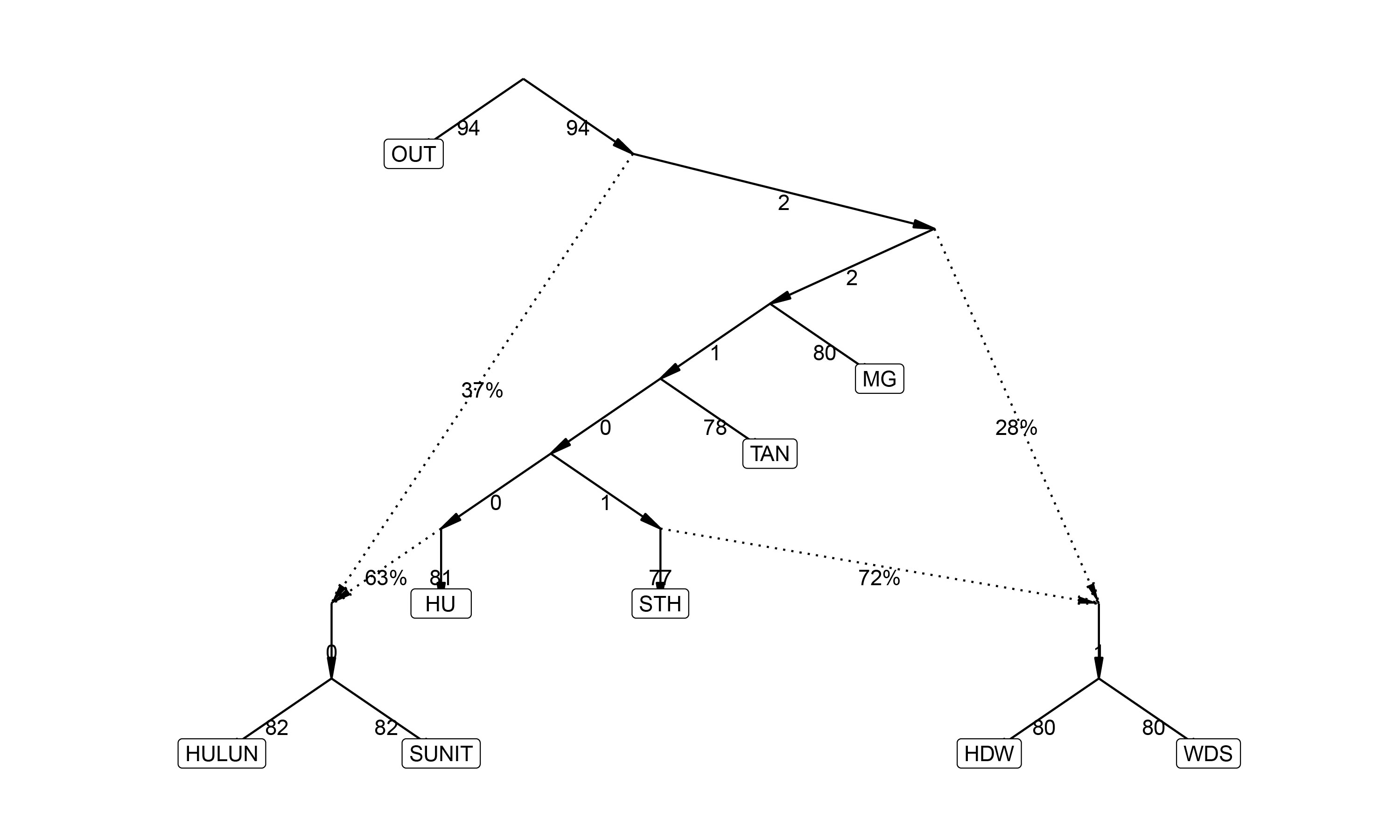


**Figure S3**. Optimal phylogenetic topology estimated by qpGraph.


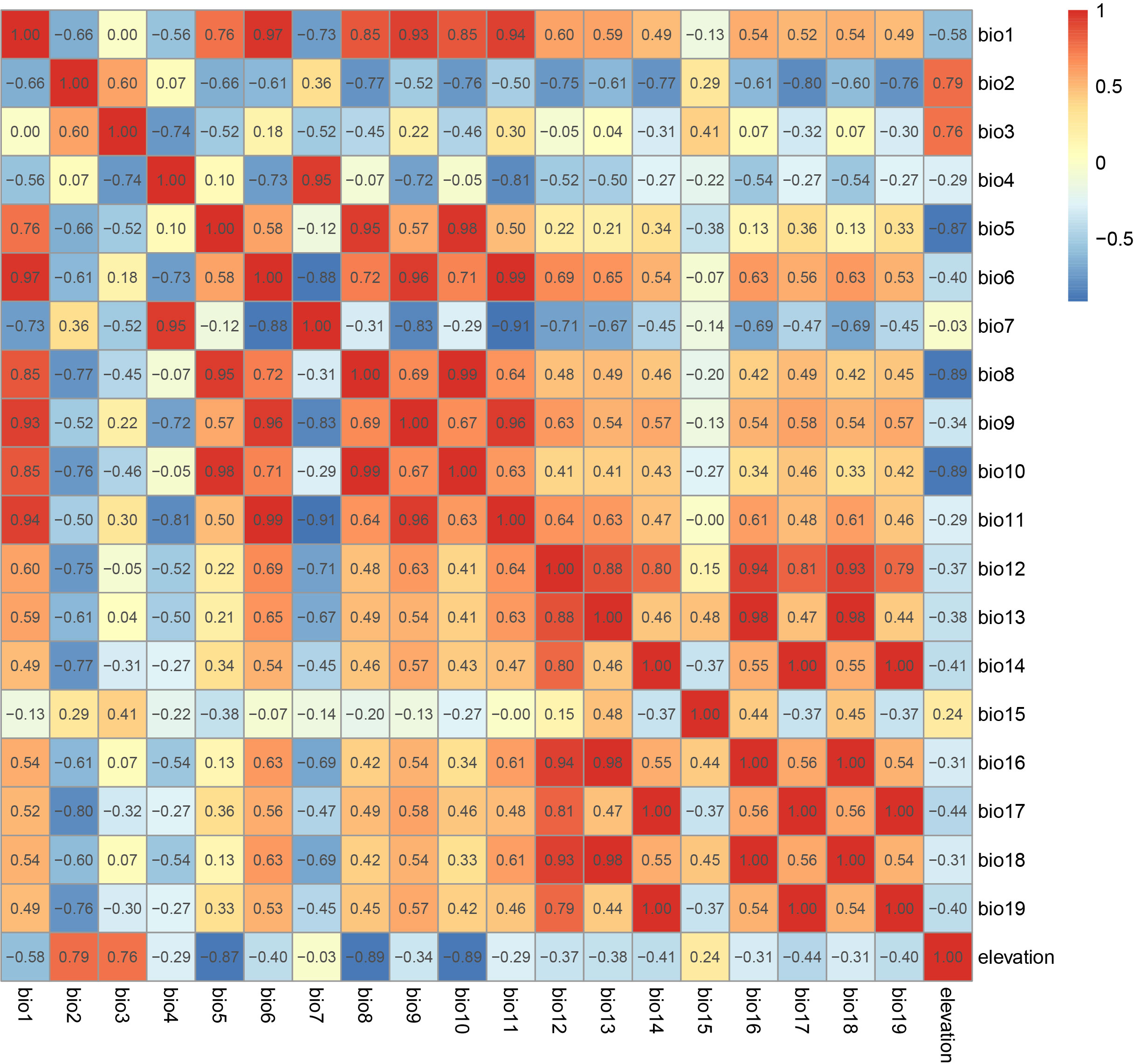


**Figure S4**. Heatmap of correlations among all bioclimatic variables. Darker colors indicate higher correlations. The numbers in the boxes represent the values of the Pearson correlation coefficients.


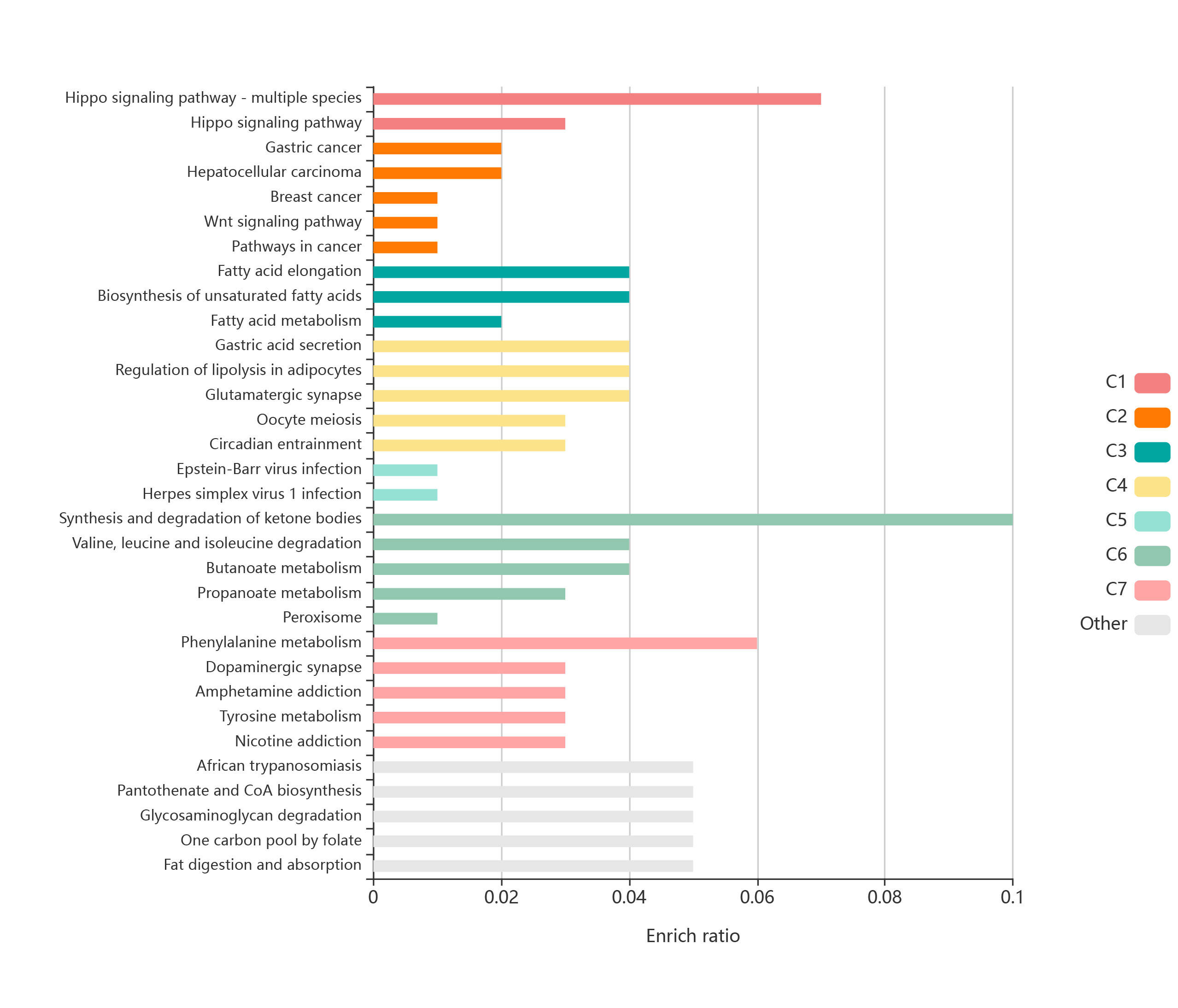


**Figure S5**. KEGG-enriched entries for significant loci in the LFMM results of BIO14. Each row represents an enriched function, and the length of the bar indicates the enrichment ratio, calculated as "input gene number"/"backgound gene number". The bar color represents different clusters.


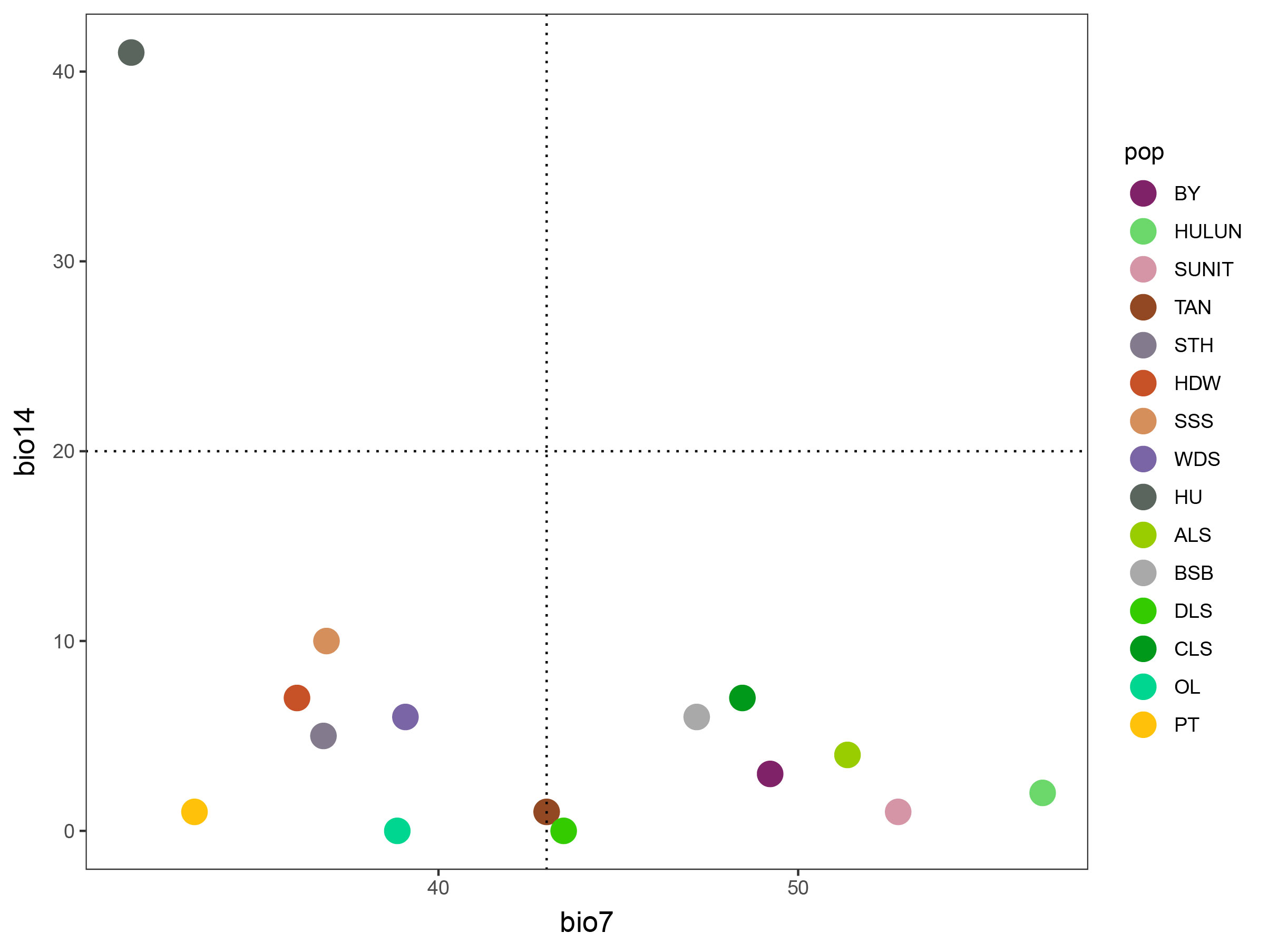


**Figure S6**. Scatterplot of groupings of different populations based on BIO7, BIO14. The populations can be clearly divided into 3 groups according to BIO14 and BIO7 based on the median. Populations in humid and stable high temperature environment include HU sheep. Populations in dry and warm environments include HDW, SSS, STH, WDS, OL, PT. The remaining populations inhabit dry and cold environments.


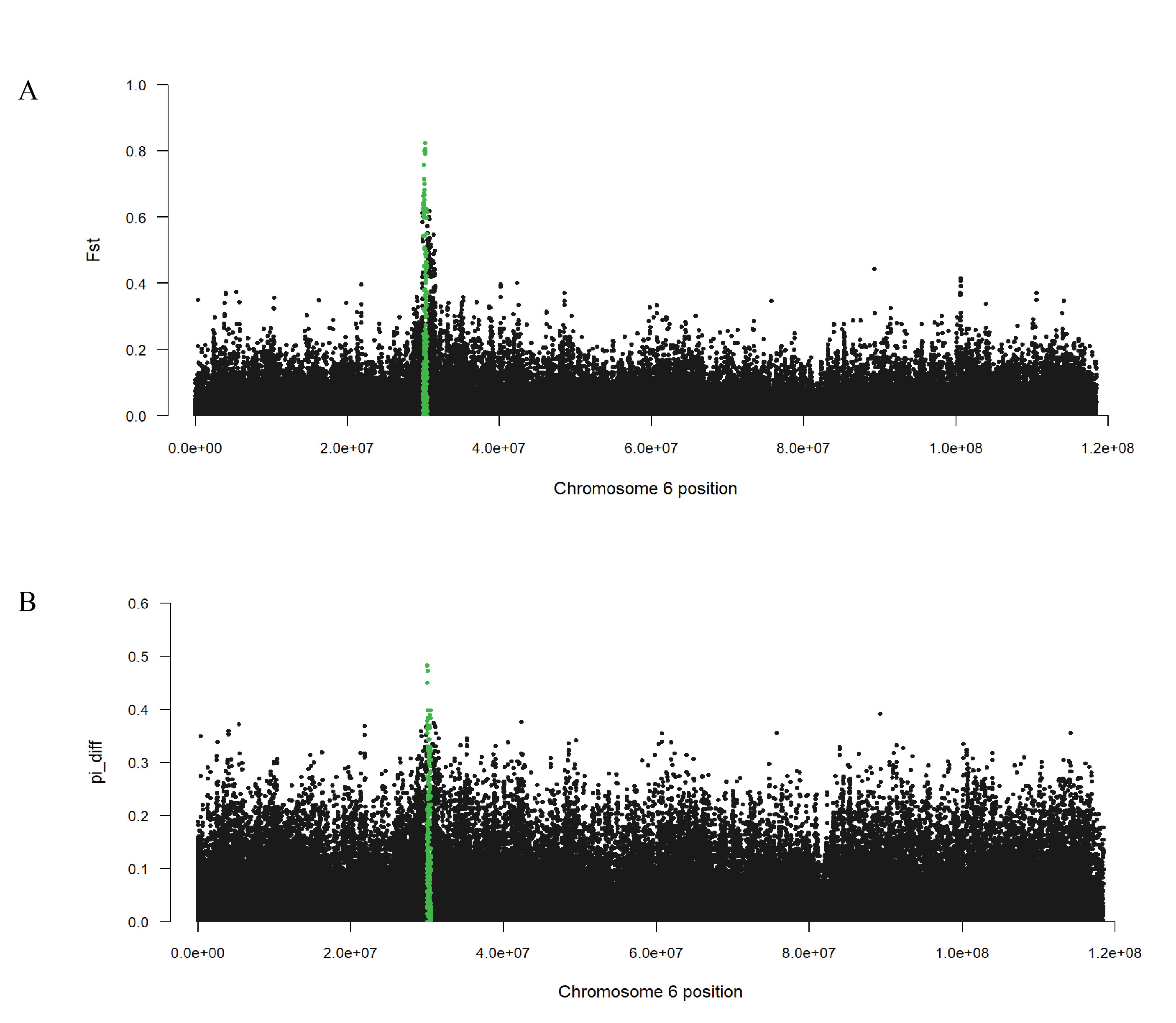


**Figure S7**. Difference in Fst (A) and Pi (B) between populations in humid and dry environment on chr6, where the green loci are located within the *BMPR1B* gene region.


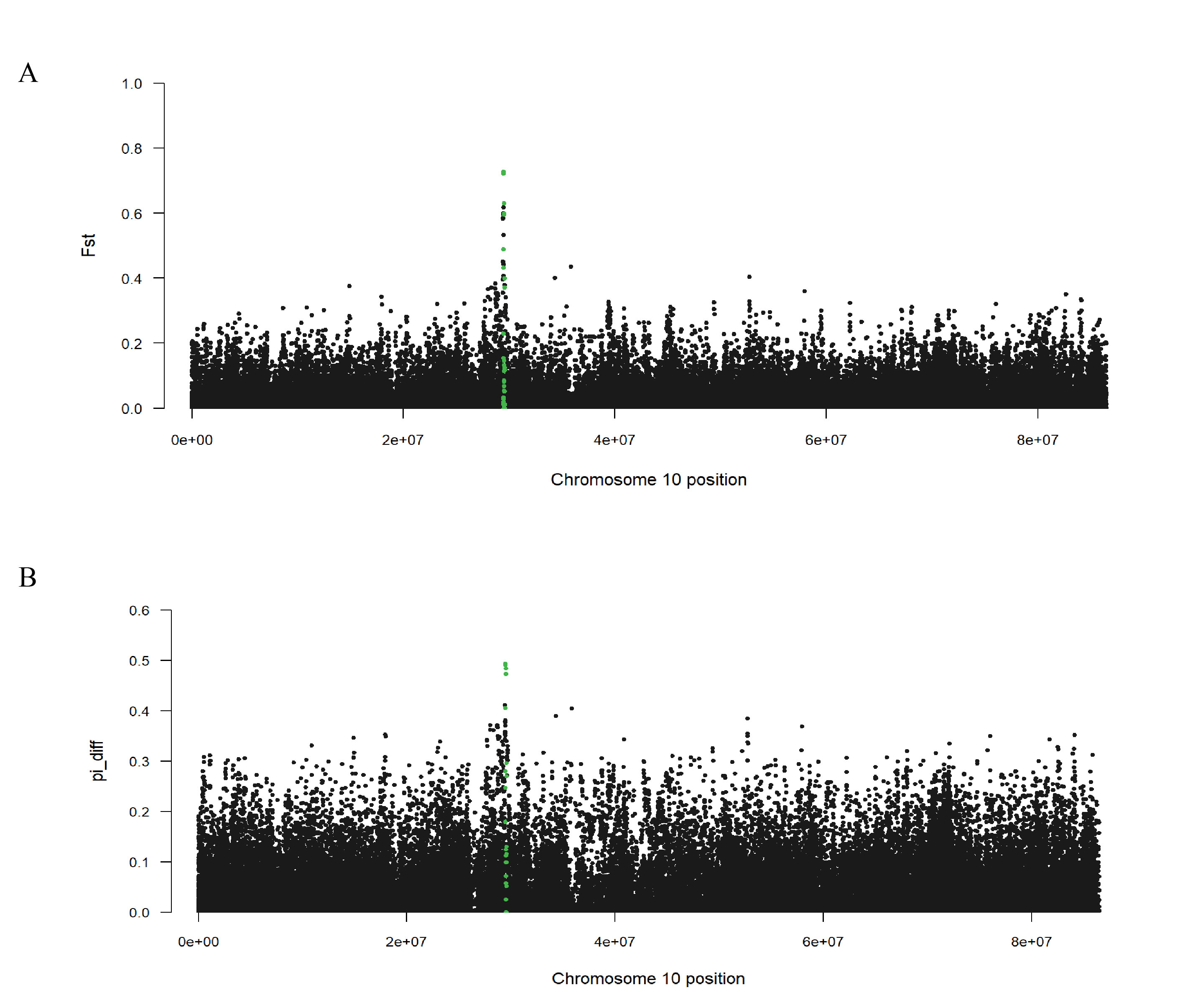


**Figure S8**. Difference in Fst (A) and Pi (B) between populations in humid and dry environment on chr10, where the green loci are located within the *RXFP2* gene region.


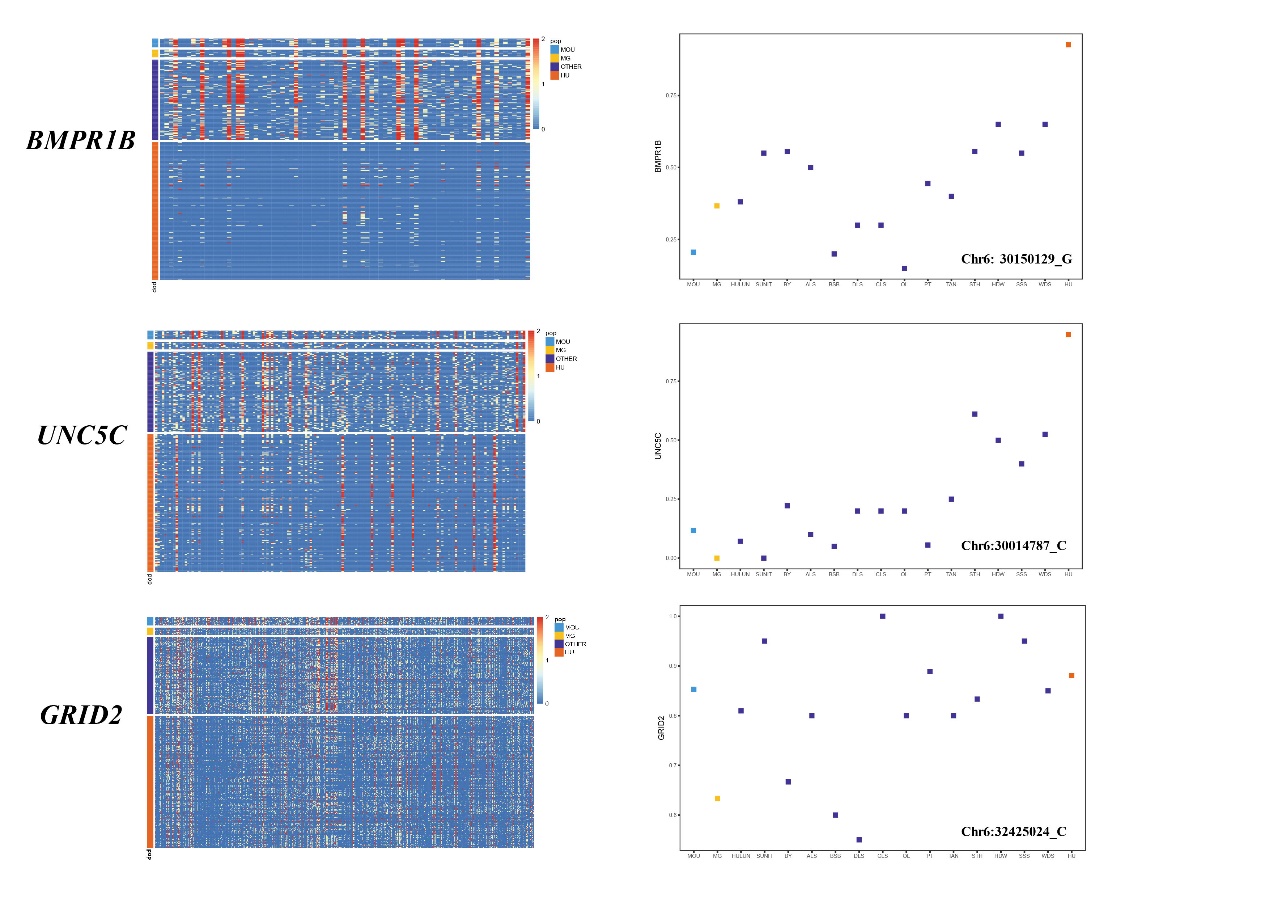


**Figure S9**. Differences in locus frequencies between wild and domesticated populations. A: On the left is a heatmap of genotypes in the *BMPR1B* region for Mouflon, Mongolian populations, domesticated populations other than Hu sheep (STH, WDS, SSS, HDW, TAN, HULUN, SUNIT, PT, OL, DLS, CLS, BY, BSB, ALS), and Hu sheep. On the right is the frequency of the locus with the most significant Fst difference in *BMPR1B*, chr6:30150129_G. B: On the left is a heatmap of genotypes in the *UNC5C* region, and on the right is the frequency of the locus with the most significant Fst difference in *UNC5C*, chr6:30014787_C. C: On the left is a heatmap of genotypes in the *GRID2* region, and on the right is the frequency of the locus with the most significant Fst difference in *GRID2*, chr6:32425024_C.


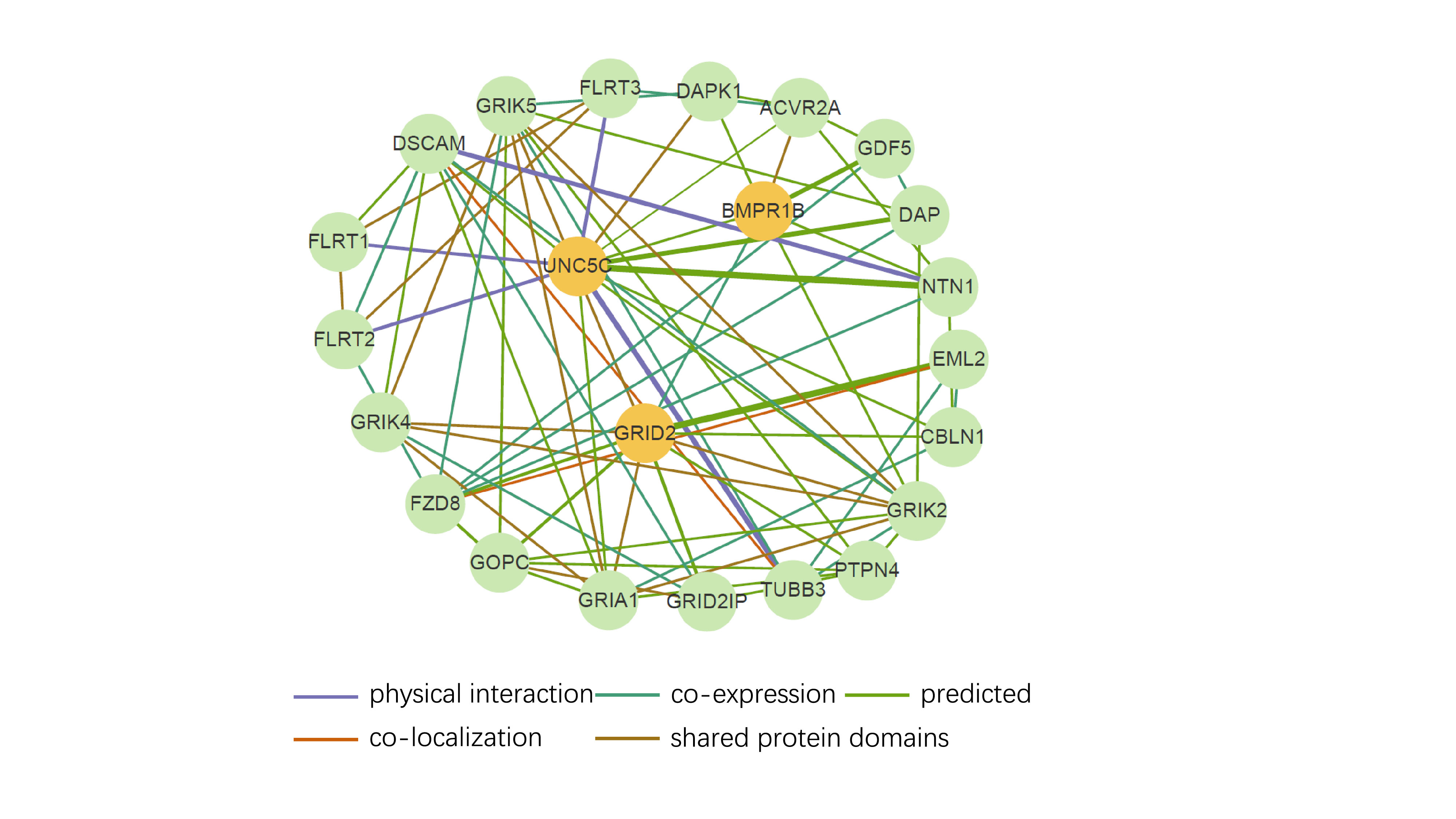


**Figure S10**. Integrative biology network comprising *BMPR1B*, *UNC5C*, *GRID2*, and other genes predicted to be possibly related. Colors of lines represent different associations between genes. Thicker lines between genes represent higher weights.
